# Protoplast-Esculin Assay as a New Method to Assay Plant Sucrose Transporters: Characterization of AtSUC6 and AtSUC7 Sucrose Uptake Activity in Arabidopsis Col-0 Ecotype

**DOI:** 10.3389/fpls.2018.00430

**Published:** 2018-04-23

**Authors:** Theresa M. Rottmann, Carolin Fritz, Anja Lauter, Sabine Schneider, Cornelia Fischer, Nina Danzberger, Petra Dietrich, Norbert Sauer, Ruth Stadler

**Affiliations:** Molecular Plant Physiology, Department of Biology, Friedrich-Alexander University Erlangen-Nürnberg, Erlangen, Germany

**Keywords:** *Arabidopsis thaliana*, sucrose, sugar transport protein, pollen tubes, radioactive uptake measurement, esculin, protoplasts, *Saccharomyces cerevisiae*

## Abstract

The best characterized function of sucrose transporters of the SUC family in plants is the uptake of sucrose into the phloem for long-distance transport of photoassimilates. This important step is usually performed by one specific SUC in every species. However, plants possess small families of several different SUCs which are less well understood. Here, we report on the characterization of AtSUC6 and AtSUC7, two members of the SUC family in *Arabidopsis thaliana*. Heterologous expression in yeast (*Saccharomyces cerevisiae*) revealed that AtSUC6_Col-0_ is a high-affinity H^+^-symporter that mediates the uptake of sucrose and maltose across the plasma membrane at exceptionally low pH values. Reporter gene analyses revealed a strong expression of *AtSUC6*_Col-0_ in reproductive tissues, where the protein product might contribute to sugar uptake into pollen tubes and synergid cells. A knockout of *AtSUC6* did not interfere with vegetative development or reproduction, which points toward physiological redundancy of AtSUC6_Col-0_ with other sugar transporters. Reporter gene analyses showed that *AtSUC7*_Col-0_ is expressed in roots and pollen tubes and that this sink specific expression of *AtSUC7*_Col-0_ is regulated by intragenic regions. Transport activity of AtSUC7_Col-0_ could not be analyzed in baker’s yeast or *Xenopus* oocytes because the protein was not correctly targeted to the plasma membrane in both heterologous expression systems. Therefore, a novel approach to analyze sucrose transporters *in planta* was developed. Plasma membrane localized SUCs including AtSUC6_Col-0_ and also sucrose specific SWEETs were able to mediate transport of the fluorescent sucrose analog esculin in transformed mesophyll protoplasts. In contrast, AtSUC7_Col-0_ is not able to mediate esculin transport across the plasma membrane which implicates that *AtSUC7*_Col-0_ might be a non-functional pseudogene. The novel protoplast assay provides a useful tool for the quick and quantitative analysis of sucrose transporters in an *in planta* expression system.

## Introduction

The complex organization of higher plants results in the coexistence of autotrophic tissues that fix CO_2_ via photosynthesis (source tissues) and heterotrophic tissues that rely on the supply with organic carbon (sink tissues) like for example roots, young leaves, meristems, and reproductive organs. The transport of fixed carbon from source to sink tissues occurs in the sieve elements of the phloem. Depending on plant species there are differences in the mode of phloem loading and the chemical structure in which carbon is transported. In plants like Arabidopsis, where sucrose represents the main transport sugar and is loaded into the phloem via an apoplastic route (Gamalei, 1989; Haritatos et al., 2000), sugar transporters are involved in at least four steps of carbon distribution: (i) release of sucrose from the mesophyll cells to the apoplast, (ii) uptake of sucrose from the apoplast into the sieve element-companion cell complex, (iii) release of sucrose into the apoplast towards symplastically isolated sink-tissues like for example pollen or embryos, and (iv) re-uptake of sucrose into these tissues (Lemoine, 2000). Whereas the first and probably also the third step are mediated by passive sucrose facilitators of the SWEET family (Chen et al., 2012), the other steps require active transporters to enable the accumulation of sucrose.

Plant genomes encode small families of SUCROSE TRANSPORTERS (SUCs or SUTs) that are members of the Major Facilitator Superfamily (Marger and Saier, 1993) and work as H^+^/sucrose-symporters (Zhou et al., 1997). In Arabidopsis, nine different AtSUCs have been identified (Lemoine, 2000; Williams et al., 2000; Sauer et al., 2004), but only AtSUC2 is required for phloem loading (Stadler and Sauer, 1996). The functions of the other AtSUCs are less well understood. AtSUC1 is involved in anthocyanin accumulation, anther dehiscence, pollen germination, and pollen tube guidance (Stadler et al., 1999; Sivitz et al., 2008). AtSUC3 is induced upon wounding and seems to deliver nutrients for repair mechanisms or remove sucrose from the apoplast to deprive potential pathogens from nutrients (Meyer et al., 2004). AtSUC5 additionally transports biotin and is needed for seed development (Ludwig et al., 2000; Baud et al., 2005; Pommerrenig et al., 2013) and AtSUC9 is associated with floral induction (Sivitz et al., 2007). AtSUC4 is the only Arabidopsis SUC localized in the tonoplast and releases sucrose from the vacuole (Schulz et al., 2011; Schneider S. et al., 2012). In Arabidopsis and other species SUCs have been shown to play roles in grain filling (Sivitz et al., 2005), sugar accumulation in fruits (Afoufa-Bastien et al., 2010; Zheng et al., 2014; Zanon et al., 2015) and other storage organs like sugar beet taproots or sugar cane stalks (Reinders et al., 2006; Jung et al., 2015). SUCs are involved in phosphate starvation responses (Lei et al., 2011), sugar signaling (Sivitz et al., 2008; Li et al., 2012) as well as interactions with symbionts (Doidy et al., 2012) and parasites (Juergensen et al., 2003; Hammes et al., 2005; Hofmann et al., 2009; Péron et al., 2017).

However, the analysis of SUC functions especially in sink tissues via *suc* mutants is complicated due to coexpression of different *SUC*s and probable redundant functions of the encoded proteins. Additionally, monosaccharide transporters of the STP family, which can import the products of sucrose cleaved by cell-wall invertases may also obscure SUC functions in tissues where both transporter types are localized (Sauer, 2007; Sturm, 1999). Furthermore, *SUC* expression is regulated by developmental cues (Truernit and Sauer, 1995), phytohormones (Chincinska et al., 2008; Feuerstein et al., 2010; Hoth et al., 2010), abiotic stress (Sakr et al., 1997; Gong et al., 2013; Jia et al., 2015), sugar state (Weber et al., 1997; Chiou and Bush, 1998; Ransom-Hodgkins et al., 2003; Price et al., 2004), temperature (Lundmark et al., 2006), and diurnal rhythms (Kühn et al., 1997). Besides altered expression, SUC activity may be modulated by phosphorylation (Roblin et al., 1998; Niittylä et al., 2007), formation of SUC heteromers (Reinders et al., 2002; Schulze et al., 2003), interaction with other proteins (Fan et al., 2009; Li et al., 2012) or indirectly via modulation of the membrane potential by regulation of the H^+^-ATPase (Sakr et al., 1997; Williams et al., 2000).

It has also been shown that the expression of some *AtSUC*s varies between ecotypes. For example, *AtSUC1* is expressed in the funicular epidermis of C24, Ler and Ws, but not in Col-0. In contrast, Col-0 pollen grains contain much higher levels of AtSUC1 than pollen of ecotypes C24, Ler and Ws (Feuerstein et al., 2010). The coding sequence of *AtSUC5* in ecotype Ler differs from the corresponding sequence in ecotype Col-0 in 8 bp leading to two amino acid exchanges (Ludwig et al., 2000). Additionally, *AtSUC9* transcripts could only be detected in unpollinated pistils of Col-0 but neither in Cvi or Ler (Leydon et al., 2017).

Previous analyses of AtSUC6 and AtSUC7 led to the assumption that *AtSUC6* and *AtSUC7* are pseudogenes coding for non-functional proteins (Sauer et al., 2004). However, sucrose uptake analysis has only been carried out by heterologous expression of *AtSUC6*_C24_, *AtSUC7*_C24_ or *AtSUC7*_Ws_ in yeast cells. Both, *AtSUC6* and *AtSUC7*, are predicted to be expressed in pollen tubes (Qin et al., 2009; Leydon et al., 2013) and it has been reported that especially genes involved in reproductive processes are highly evolutionary plastic both in their sequence and their expression patterns (Leydon et al., 2017). In fact, both genes contain many ecotype specific amino acid exchanges (Sauer et al., 2004). Furthermore, in some cases yeast cells are not suitable as heterologous expression systems for the analysis of transporter activities. The evolutionary distance between plants and yeasts may lead to the problem that the plant protein is not targeted to the correct membrane in yeast (Barker et al., 2000; Schneider S. et al., 2012) or lacks essential protein modifications and therefore does not show its normal activity in the heterologous system.

In the present paper, we present the detailed analysis of AtSUC6 and AtSUC7 of ecotype Col-0. *AtSUC6*_Col-0_ is expressed in the vasculature and reproductive cells, *AtSUC7*_Col-0_ in roots and pollen tubes. The transport characteristics of AtSUC6_Col-0_ were analyzed in *S. cerevisiae* revealing that it is a H^+^/sucrose-symporter with exceptional high affinity for sucrose and a low pH optimum. AtSUC7_Col-0_ characterization in yeast was not possible due to mistargeting of the protein to internal membranes. A novel protoplast assay with the sucrose analog esculin enables the analysis of sucrose transporter activities in the plant system and revealed that AtSUC7_Col-0_ is not able to transport esculin due to the lack of two conserved amino acids. T-DNA insertion lines for *AtSUC6*_Col-0_ and *AtSUC7*_Col-0_ were characterized but did not show any phenotypical differences compared to wild type (WT) plants. Potential physiological functions for AtSUC6 are discussed.

## Materials and Methods

### Strains, Growth Conditions, and Genotyping

*Arabidopsis thaliana* (L.) HEYNH. ecotypes Col-0, C24 and Wassilewskija were grown in the greenhouse in potting soil or under long-day conditions (16 h light/8 h dark) at 22°C and 60% relative humidity in a phytochamber. Plants for the generation of protoplasts were cultivated under a short-day regime (8 h light/16 h dark). For analyses of seedlings or roots, surface-sterilized seeds were cultivated on MS plates (Murashige and Skoog, 1962) under long-day conditions at 22°C. The T-DNA insertion lines *Atsuc6.1* (SALK_132450; Alonso et al., 2003)*, Atsuc6.2* (SALK_108259; Alonso et al., 2003)*, Atsuc6.3* (SM_3.18900; Tissier et al., 1999), *Atsuc6.4* (SM_3.41113; Tissier et al., 1999), *Atsuc7.1* (GABI_054G04; Kleinboelting et al., 2012), *Atsuc7.2* (SAIL_221_C05; Sessions et al., 2002), and *Atsuc7.3* (GABI_374G11; Kleinboelting et al., 2012) were obtained from the Nottingham Stock Centre^1^. The *Attmt1/tmt2* double mutant line (Wormit et al., 2006) was kindly provided by Ekkehard Neuhaus (Division of Plant Physiology, University of Kaiserslautern). The companion cell marker lines pMH5a and pEPS1 have been described by Imlau et al. (1999) and Stadler et al. (2005b), respectively. The primers used for genotyping are listed in Supplementary Table 2. Segregation analyses of the *Atsuc6.3* and *Atsuc7.3* alleles were performed by PCR-based genotyping with the respective primer pairs. The positions of the T-DNA insertions were reviewed by sequencing of PCR products obtained with the primer pair for the mutant allele (Supplementary Table 2). Transformations of *Arabidopsis thaliana* were performed via floral dip with *Agrobacterium tumefaciens* SMITH AND TOWNSEND strain GV3101 (Holsters et al., 1980; Clough and Bent, 1998). *Escherichia coli* (MIGULA) CASTELLANI AND CHALMERS strain DH5α (Hanahan, 1983) was used for all cloning steps. Heterologous expression analyses were performed in *Saccharomyces cerevisiae* MEYEN *ex* E.C. HANSEN strains CSY4000 (Rottmann et al., 2016) or SEY2102 (Emr et al., 1983). SEY2102 containing the sucrose transporter Srt1 (Wahl et al., 2010) was used as a positive control in uptake experiments.

### RNA Isolation and RT-PCR

Total RNA was isolated from different Arabidopsis tissues with TRIzol reagent (Invitrogen). RNA isolation from pollen was performed as described (Rottmann et al., 2016). The QuantiTect^®^ Reverse Transcription Kit (Qiagen) was used for reverse transcription reactions. Detection of *AtSUC6*_Col-0_ and *AtSUC7*_Col-0_ transcripts was carried out by PCR with the primer pairs listed in Supplementary Tables 1, 3. A PCR with primers for *ACTIN2c* was performed as a positive control.

### Cloning of Reporter Gene Constructs for *AtSUC*6_Col-0_ and *AtSUC7*_Col-0_

For the p*AtSUC6:AtSUC6*g-reporter plants a 4,478-bp fragment including the genomic sequence of *AtSUC6*_Col-0_ and 2,547 bp upstream of the start ATG was amplified with the primer pair AtSUC6-2547f+CACC and AtSUC6c+1476r (Supplementary Table 4), cloned into pENTR/D-TOPO (Invitrogen) and inserted upstream of the *GUS*- or *GFP*::nos terminator box by LR-reaction in pBASTA-GUS or pBASTA-GFP (Rottmann et al., 2016) yielding plasmids pTR314 and pTR315, respectively. For reporter plants expressing *GUS* or *GFP* fusions of *AtSUC7*_Col-0_ under the control of the native promoter a 3,826-bp fragment including 1,896 bp upstream of the start ATG was amplified with primers AtSUC7-1896f+CACC and AtSUC7c+1473r (Supplementary Table 4) and cloned into pENTR/D-TOPO (Invitrogen). The complete p*AtSUC7:AtSUC7*g sequence was finally cloned into pBASTA-GUS or pBASTA-GFP by LR reaction yielding plasmids pTR3 and pTR4, respectively. For reporter plants expressing *GUS* without the genomic sequence of *AtSUC7* under the control of the *AtSUC7*_Col-0_ promoter the 1,896-bp fragment upstream of the start ATG was amplified with primers AtSUC7-1896f+*Sbf*I and AtSUC7-1r+*Asc*I (Supplementary Table 4) and used to exchange the *35S* promoter of the Gateway^®^ vector pMDC43 (Curtis and Grossniklaus, 2003) in front of the attachment site AttR1 via the added *Sbf*I/*Asc*I sites. The coding sequence for *GUS* was then inserted via LR reaction from pENTR-GUS (Invitrogen) yielding plasmid pTR95.

### Cloning of *GFP* Fusion Constructs for Protoplast Transformation

For the subcellular localization analysis of AtSUC6_Col-0_ and AtSUC7_Col-0_, fusion constructs of the respective coding sequences with *GFP* under the control of the *35S* promoter were generated. The coding sequence of *AtSUC6*_Col-0_ was amplified in two parts with the primer pairs AtSUC6-54f/AtSUC6c+622r and AtSUC6c+581f/AtSUC6c+1521r (Supplementary Table 6) from silique cDNA and cloned into pJET1.2blunt (Thermo Scientific). The primers AtSUC6-54f and AtSUC6c+1521r were designed to bind in the UTR due to the homology of SUCs at the ends of their coding sequences. The resulting plasmids served as templates for the amplification with the primer pairs AtSUC6c+1f+CACC/AtSUC6c+622r and AtSUC6c+518f/AtSUC6c+1479r+*Asc*I (Supplementary Table 6). Both fragments were finally assembled in pENTR-D/TOPO (Invitrogen) via the internal *Bgl*II and the *Asc*I site attached by PCR and inserted into pMDC43 (Curtis and Grossniklaus, 2003) via LR reaction yielding plasmid pFC12. For the *AtSUC6*_Col-0_*-GFP* fusion construct the reverse primer lacking the stop codon was used and the fragment was inserted into pMDC83 (Curtis and Grossniklaus, 2003) yielding plasmid pFC13. The *AtSUC7*_Col-0_ coding sequence was amplified from pollen tube cDNA with primers AtSUC7-32f/AtSUC7c+1507r (Supplementary Table 6) due to homology and reamplified with AtSUC7c+1f+*Bsp*HI/AtSUC7c+1473r+*Bsp*HI (Supplementary Table 6). The resulting fragments were then inserted into the *Nco*I site of pCS120 (Dotzauer et al., 2010) or pSS87 (Schneider S. et al., 2012), yielding plasmids pTR57 (*AtSUC7c-GFP*) and pTR58 (*GFP-AtSUC7c*). To insert a spacer between *AtSUC7c* and *GFP* primers AtSUC7c+1f+CACC and AtSUC7c+1476r or AtSUC7c+1473r (Supplementary Table 6) were used to amplify *AtSUC7*_Col-0_ with or without the stop codon from pTR57. Both PCR products were ligated into pENTR/D-TOPO (Invitrogen) and then inserted into pMDC83 (Curtis and Grossniklaus, 2003) for *AtSUC7c-GFP* or pMDC43 (Curtis and Grossniklaus, 2003) for *GFP-AtSUC7c*, yielding plasmids pTR73 and pTR72, respectively. The CDS of *AtSUC7*_Ws_ from ecotype Wassilewskija was amplified with primers AtSUC7c+1f+CACC and AtSUC7c+1507r (Supplementary Table 6) and cloned into pMDC43 yielding plasmid pTR253.

Further constructs for expression in protoplasts were generated by amplification of the CDS from existing templates with the primer pairs listed in Supplementary Table 6. Primers for *AtSUC2c*, *AtSUC9c*, *SWEET4c*, and *SWEET10c* attached *Nco*I or *Bsp*HI sites to both ends of the amplified sequences that were finally inserted into pSS87 or pCS120 yielding the constructs listed in Supplementary Table 6. *AtSUC3c* and *AtSUC8c* were extended for CACC in front of the start ATG, cloned into pENTR-D/TOPO (Invitrogen) and brought into pMDC43 or pMDC83 (Curtis and Grossniklaus, 2003) by LR reactions. The resulting plasmids are listed in Supplementary Table 6.

Site-directed mutagenesis with mismatching primers (Supplementary Table 6) was used to replace the proline at position 67 in *AtSUC7c* with serine. Similarly, arginine was replaced by glycine at position 436. Both PCR fragments were subcloned into pJET1.2blunt (Thermo Scientific). For the construct carrying both point mutations the *N*-terminal sequence of *AtSUC7*c_P67S_ was used to replace the *N*-terminal sequence of *AtSUC7*c_R436G_ via the internal *Mlu*I site and the *Pst*I site of the vector. All three sequence variants were finally cloned into pENTR/D-TOPO and inserted into pMDC43 via LR reaction yielding the plasmids listed in Supplementary Table 6. Site-directed mutageneses of *AtSUC2c* and *AtSUC5c* were performed in the same way using the mismatching primers listed in Supplementary Table 6. The resulting PCR products were cloned into pJET1.2blunt (Thermo Scientific) and both mutations were united in one plasmid each by digestion with *Mlu*I/*Xho*I (*AtSUC2c*) or *Mfe*I/*Hind*III (*AtSUC5c*). Insertions into pSS87 (Schneider S. et al., 2012) via *Nco*I led to the plasmids for protoplast transformation listed in Supplementary Table 6.

### Functional Characterization of SUCs by Heterologous Expression in Baker’s Yeast

The coding sequences of *AtSUC6*_Col-0_ and *AtSUC7*_Col-0_ were amplified from pCF12 and pTR73 with primer pairs that introduced a *Not*I site on both sides of the PCR products as well as the sequence 5′-AAGCTTGTAAAAGAA-3′ (part of the *STP1* 5′UTR) (Stadler et al., 1995) upstream of the start codon (Supplementary Table 5). The fragments were ligated into the *Not*I site of the vector NEV-N (Sauer and Stolz, 1994) in both sense and antisense orientation, yielding plasmids pFC14 *(AtSUC6c sense*), pFC19 (*AtSUC6*c antisense), pTR10 (*AtSUC7c sense*), and pTR11 (*AtSUC7c antisense*). The *AtSUC6* constructs were then used for lithium acetate-mediated transformation (Soni et al., 1993) of *S. cerevisiae* CSY4000 (Rottmann et al., 2016), yielding strains TRY1039 (*AtSUC6c sense*) and TRY1040 (*AtSUC6c antisense*). *AtSUC7* constructs were transformed into *S. cerevisiae* strain SEY2102 (Emr et al., 1983), yielding TRY1001 (*AtSUC7c sense*), and TRY1002 (*AtSUC7c antisense*). Transport tests with ^14^C-labeled sucrose were performed as described (Sauer and Stadler, 1993). *AtSUC7*_Col-0_ was additionally amplified with primers attaching *Bsp*HI sites to both ends of the fragment (Supplementary Table 5) and ligated into NEV-CGFP (Rottmann et al., 2018) and NEV-NGFP (see below) linearized with *Nco*I for analyses of the subcellular localization in yeast. The transformation of SEY2102 led to strains TRY1006 (*AtSUC7c*-NEV-NGFP) and TRY1008 (*AtSUC7c*-NEV-CGFP).

### Generation of the Yeast Expression Vector NEV-NGFP

For the analysis of protein localization in yeast cells a new expression vector was generated that allows insertion of a CDS downstream of *GFP* via *Nco*I. To this end *Pci*I/*Nco*I were attached to *GFP* by PCR as described (Schneider S. et al., 2012). The restriction sites were used to insert *GFP* into the *Nco*I site of NEV-Nco (Nieberl et al., 2017) leading to the new plasmid NEV-NGFP.

### Cloning of a *AtSUC7*_Col-0_ Construct for Expression in *Xenopus* Oocytes

For expression of *AtSUC7_Col-0_* in *Xenopus* oocytes two new Gateway^®^-compatible destination vectors were generated. The Gateway^®^-cassette from pB2GW7 (Karimi et al., 2002) was excised with *Eco*RV and inserted into *Sma*I-digested pGEMHE (Liman et al., 1992) via blunt end cloning yielding pGEMHE-GW. For the construction of pGEM-GFP, the *GFP* coding sequence was amplified from pMDC83 (Curtis and Grossniklaus, 2003) with primers SalI_attR2_f (5′-GTCGACCATAGTGACTGGATATGTTG-3′) and mGFP6_HindIII_r (5′-AAGCTTTTAGTGGTGGTGGTGGTGGTG-3′) and ligated into pGEMHE-GW via the *Sal*I and *Hind*III sites attached by PCR. The *AtSUC7*_Col-0_ CDS from the respective entry clone (see above) was then inserted into pGEMHE-GW and pGEM-GFP by LR reaction yielding expression vectors pTR66 and pTR65, respectively.

cRNA was obtained by *in vitro* transcription using the mMESSAGE mMACHINE T7 Transcription Kit (Ambion Inc., Huntington, United Kingdom). Oocytes were injected as described (Schneider et al., 2007) and incubated for 3–4 days prior to confocal analyses and uptake measurements as described in Schneider et al. (2007).

### Pollen Germination

Pollen germination *in vitro* and semi*-in vivo* for the analyses of reporter genes and pollen tube growth rates was carried out as described (Rottmann et al., 2016). Pollen tube length was measured with a self-written Python script (Python Software Foundation, Beaverton, OR, United States) and plotted with Matplotlib (Hunter, 2007). Matplotlib was also used for all other graphs.

### Protoplast Esculin Uptake Assay

Leaf mesophyll protoplasts from Col-0 plants were generated as described (Drechsel et al., 2011). After transformation via the polyethylene glycol method (Abel and Theologis, 1994) protoplasts were kept in 12-well-plates for approximately 24 h at 22°C in the dark. Esculin in W5 buffer was added to 500 μl of protoplast suspension in W5 (pH 5.6) in a 1.5 ml cup to a final concentration of 1 mM. After gently inverting the cup, protoplasts were incubated for 40 min at room temperature in the dark. During this time protoplasts descended to the bottom of the cup and prior to microscopy the supernatant was removed and replaced with 500 μl of W5 without esculin.

### Microscopy

Images of GFP-reporter plants, *GFP* expressing yeast strains and protoplasts were taken on a Leica 765 TCS SPII confocal laser scanning microscope (Leica Microsystems) and processed with Leica Confocal Software 2.5. A 488-nm argon laser was used for excitation of GFP, chlorophyll autofluorescence and propidium iodide. The 415-nm diode was used for the excitation of esculin fluorescence. Detection windows ranged from 497 to 526 nm for GFP, from 682 to 730 nm for chlorophyll autofluorescence, from 589 to 684 nm for propidium iodide, and from 424 to 469 nm for esculin. Images of GFP and esculin fluorescence for the esculin uptake assay were taken in a sequential mode. Fluorescence intensities of GFP and esculin were quantified in three regions of a defined size in each protoplast image using ImageJ 1.50b (Schneider C.A. et al., 2012). Images of GUS plants were taken with a Zeiss Axioskop (Carl Zeiss Jena GmbH) or with a Leica MZFLIII stereomicroscope (Leica Microsystems). For cross-sections stained roots were embedded into Technovit as described (Beeckman and Viane, 2000; Ühlken et al., 2014) and sections were cut using a Leica RM2135 rotary microtome. Image processing was done using the analySIS Doku 3.2 software (Soft Imaging System, Münster), ImageJ 1.50b (Schneider C.A. et al., 2012) and GIMP2.8^2^.

## Results

### Expression and Sequence Analysis of *AtSUC6* and *AtSUC7*

**FIGURE 1:**
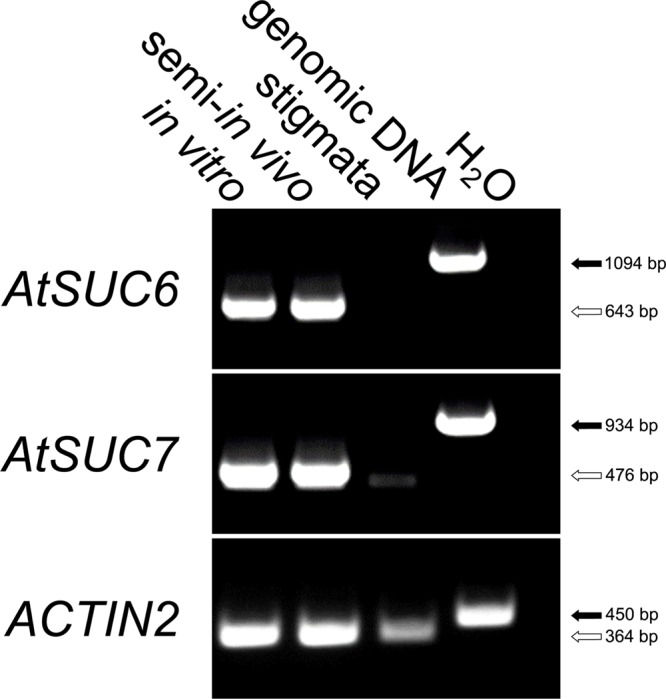
RT-PCR based expression analysis of *AtSUC6*_Col-0_ and *AtSUC7*_Col-0_ in pollen tubes. Comparison of *AtSUC6*_Col-0_ and *AtSUC7*_Col-0_ expression in *in vitro* germinated pollen tubes, pollen tubes grown through the stigma (semi-*in vivo*) and virgin stigmata with gene specific primers (Supplementary Table 1). Arrows indicate the size of PCR products derived from reverse-transcribed mRNA (white) and genomic DNA (black). The presence of cDNA in each sample was confirmed with *ACTIN2* specific primers (Supplementary Table 1).

RT-PCR analyses of genes for sucrose transporters expressed in pollen tubes indicated that both, *AtSUC6* and *AtSUC7* are strongly expressed in pollen tubes grown *in vitro* or semi-*in vivo*, whereas no transcripts could be amplified from stigmata-derived cDNA (**Figure 1**). In an earlier publication (Sauer et al., 2004) both genes have been described as pseudogenes due to splice variants leading to truncated proteins. However, sequencing of the full-length *AtSUC7*_Col-0_ coding sequence derived from pollen tube mRNA verified the predicted exon/intron structure as annotated in the TAIR10 genome. Splice variants lacking the second exon as described in Sauer et al. (2004) could not be detected and resequencing of a PCR product amplifying the *AtSUC7*_Col-0_ genomic sequence confirmed the presence of a correct splice consensus sequence at the 3′ end of the second intron. *AtSUC7*_Col-0_ consists of three exons separated by two introns as most other *SUCs*. The encoded protein consists of 491 amino acids, has a molecular mass of 52.87 kD and an isoelectric point of 8.24. Hydropathy analysis predicts the protein to have 12 transmembrane domains, a further common feature of all SUCs analyzed so far.

A comparison of the *AtSUC6*_Col-0_ coding sequence with the published TAIR10 sequence also verified the predicted exon/intron structure with three exons and two introns. Interestingly, sequencing of the *AtSUC6* genomic DNA and cDNA of C24 revealed that the conserved start ATG is missing in this ecotype. Therefore, the encoded AtSUC6_C24_ protein displays a deletion of the 32 *N*-terminal amino acids. The C24-derived sequence exhibits an additional deletion of two amino acids in the last part of the sequence and five single amino acid substitutions in comparison to Col-0. The AtSUC6_Col-0_ protein has a predicted molecular mass of 52.73 kD and an isoelectric point of 8.52. Like for all other SUCs, hydropathy analyses indicate that the AtSUC6_Col-0_ protein is a membrane protein with 12 transmembrane domains. In Arabidopsis, the SUC family consists of nine members and is subdivided into three groups. AtSUC6 and AtSUC7 show a high sequence homology to each other with 91/95% identical/similar amino acids and both are closely related to AtSUC8 (AtSUC6-AtSUC8: 92/96% identity/similarity; AtSUC7-AtSUC8: 96/98% identity/similarity). The next closest relative to this group of closely related proteins is AtSUC9. This indicates that AtSUC6 and AtSUC7 belong to the group of type I sucrose transporters like their closest relatives AtSUC8 and AtSUC9 together with AtSUC1, AtSUC2, and AtSUC5. Due to the extremely high sequence identity of all AtSUCs and especially of *AtSUC6*, *AtSUC7*, *AtSUC8*, and *AtSUC9* (up to 94%) even at the DNA level, RT-PCR conditions had to be very constrictive to avoid unspecific binding of the primer pairs. Therefore, further expression analysis of *AtSUC6*_Col-0_ and *AtSUC7*_Col-0_ by RT-PCR was not performed as the amplification of other *AtSUC* transcripts or the loss of the specific signal in tissues with a low expression level of *AtSUC6* or *AtSUC7* could not be excluded. Instead, the exact expression patterns of *AtSUC6*_Col-0_ and *AtSUC7*_Col-0_ were analyzed with reporter plants.

### Reporter Gene Analyses of *AtSUC6*_Col-0_ and *AtSUC7*_Col-0_ Expression

To analyze the expression of *AtSUC6*_Col-0_ and *AtSUC7*_Col-0_ in more detail p*SUC*:*SUC*g-*GUS* and -*GFP* lines were generated. Plants expressing *AtSUC6*g:*GUS* or *AtSUC6*g:*GFP* from a 2,547-bp promoter fragment of *AtSUC6*_Col-0_ were generated by Agrobacteria-mediated transformation of Arabidopsis Col-0 with plasmids pTR314 (GUS) or pTR315 (GFP). At least eight different lines of the resulting BASTA resistant plants were analyzed in detail.

**FIGURE 2:**
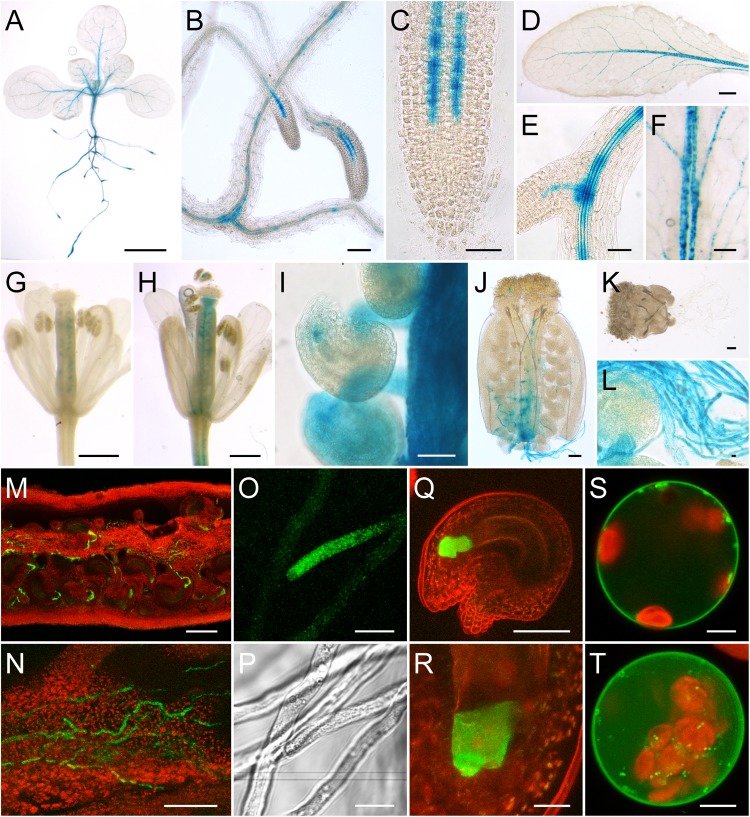
Analysis of p*AtSUC6*:*AtSUC6*g-reporter plants and subcellular localization of AtSUC6_Col-0_. **(A–L)** Histochemical detection of β-glucuronidase activity in Arabidopsis Col-0 expressing *AtSUC6*g-*GUS* under the control of the native *AtSUC6*_Col-0_ promoter. **(A)** Two-week-old seedling with GUS staining in the vascular tissue of roots, hypocotyl and leaves. **(B)** Lateral root tips of a 2-week-old seedling. **(C)** Root tip with GUS activity in the protophloem at higher magnification. **(D)** Rosette leaf. **(E)** GUS staining in the vasculature of the root differentiation zone. **(F)** Patchy GUS pattern in the vasculature of a source leaf. **(G)** Unpollinated flower in early stage 13 (all flower stages according to Smyth et al., 1990) with GUS signal in the ovules. **(H)** Pollinated flower of stage 14. **(I)** Peeled ovary of a stage-14 flower. **(J)** Pollen tubes grown semi-*in vivo* through a WT stigma and a part of the transmitting tract. **(K)** Pollen tubes germinated semi-*in vivo* on a WT stigma. **(L)** Pollen tubes at higher magnification. **(M–R)** Detection of GFP fluorescence (green) in p*AtSUC6*:*AtSUC6*g-*GFP* reporter plants. Chlorophyll autofluorescence is given in red. **(M)** Peeled ovary with GFP fluorescence in pollen tubes. **(N)** Pollen tubes growing in the transmitting tract of a peeled ovary. **(O)** Tip of a pollen tube grown semi-*in vivo*. **(P)** Bright field image of **(O)**. **(Q)** Maximum projection of an excised ovule stained with propidium iodide (red) with GFP fluorescence in the synergids. **(R)** Synergid cells at higher magnification. **(S,T)** Single optical section **(S)** and maximum projection **(T)** of a protoplast expressing *AtSUC6*c*-GFP* under the control of the *35S* promoter. Scale bars: 2.5 mm in **(A,D)**; 100 μm in **(B,M)**; 50 μm in **(C,E,I,J,K,N,Q)**; 1 mm in **(F)**; 500 μm in **(G,H)**; 10 μm in **(L,O,P,R)**; 5 μm in **(S,T)**.

In 2-week-old seedlings, GUS staining was detected in the vasculature of roots, hypocotyls, and leaves (**Figure 2A**). Especially root tips (**Figures 2B,C**) and the base of young lateral roots (**Figures 2B,E**) showed a dark blue staining. Expression of *AtSUC6:GUS* in root tips was restricted to cell rows in the stele, possibly the protophloem (**Figure 2C**). GUS staining could be observed in cotyledons and primary leaves (**Figure 2A**) as well as in fully developed rosette leaves (**Figure 2D**). Here the staining was strongest in the midrib and in major veins. The punctate pattern of the staining in the vasculature (**Figures 2C,F**) indicates that the expression of *AtSUC6:GUS* is restricted to single cells or a specific cell type of the vascular tissue. In flowers with emerged petals (early stage 13; Smyth et al., 1990) GUS staining was detectable in the ovules prior to pollination (**Figure 2G**). When ovaries were peeled, it became visible that the blue staining in ovules was restricted to a small area near the micropyle **Figure 2I**). Pollinated flowers of stage 14 showed additional staining in the transmitting tract (**Figure 2H**). Strong GUS activity was detectable in pollen tubes grown semi-*in vivo* through the stigma and the upper part of the ovary (**Figures 2J,L**). Interestingly, no GUS staining was detectable in pollen tubes grown *in vitro* or through a stigma that had been excised from the ovary (**Figure 2K**). This indicated that *AtSUC6:GUS* expression or translation is induced during the pollen tube’s growth through the transmitting tract of the ovary. The intense GUS activity in pollen tubes (**Figures 2J,L**) and the absence of GUS staining in the transmitting tract of unpollinated pistils (**Figure 2G**) indicated that the GUS staining observed in the transmitting tract of open flowers (**Figure 2H**) did not originate from the maternal tissue itself but from pollen tubes. This was also confirmed by the analysis of p*AtSUC6*:*AtSUC6*g-*GFP* lines, where GFP fluorescence in the transmitting tract of pollinated flowers clearly originated from pollen tubes (**Figures 2M,N**). GFP fluorescence was also observed in pollen tubes grown through the upper part of a pistil in a semi-*in vivo* pollination assay (**Figures 2O,P**). Plants expressing *GFP* as a reporter gene furthermore showed that *AtSUC6*_Col-0_ expression in ovules is restricted to synergid cells (**Figures 2Q,R**). Expression of *AtSUC6*_Col-0_ in the vascular tissue could not be analyzed in the GFP reporter plants due to a weaker expression compared to flowers and strong chlorophyll autofluorescence in leaves. To analyze the subcellular localization of AtSUC6_Col-0_, *C*- and *N*-terminal fusion constructs with *GFP* were expressed in Arabidopsis mesophyll protoplasts under the control of the *35S* promoter. The *N*-terminal fusion of GFP to AtSUC6_Col-0_ probably interfered with protein targeting leading to most fusion proteins remaining in the endomembrane system (Supplementary Figure 4A). The *C*-terminal GFP-AtSUC6_Col-0_ fusions labeled the plasma membrane in single optical sections (**Figure 2S**) and maximum projections (**Figure 2T**), with only some fusion proteins remaining in the endomembrane system, indicating that AtSUC6 is a plasma membrane protein.

**FIGURE 3:**
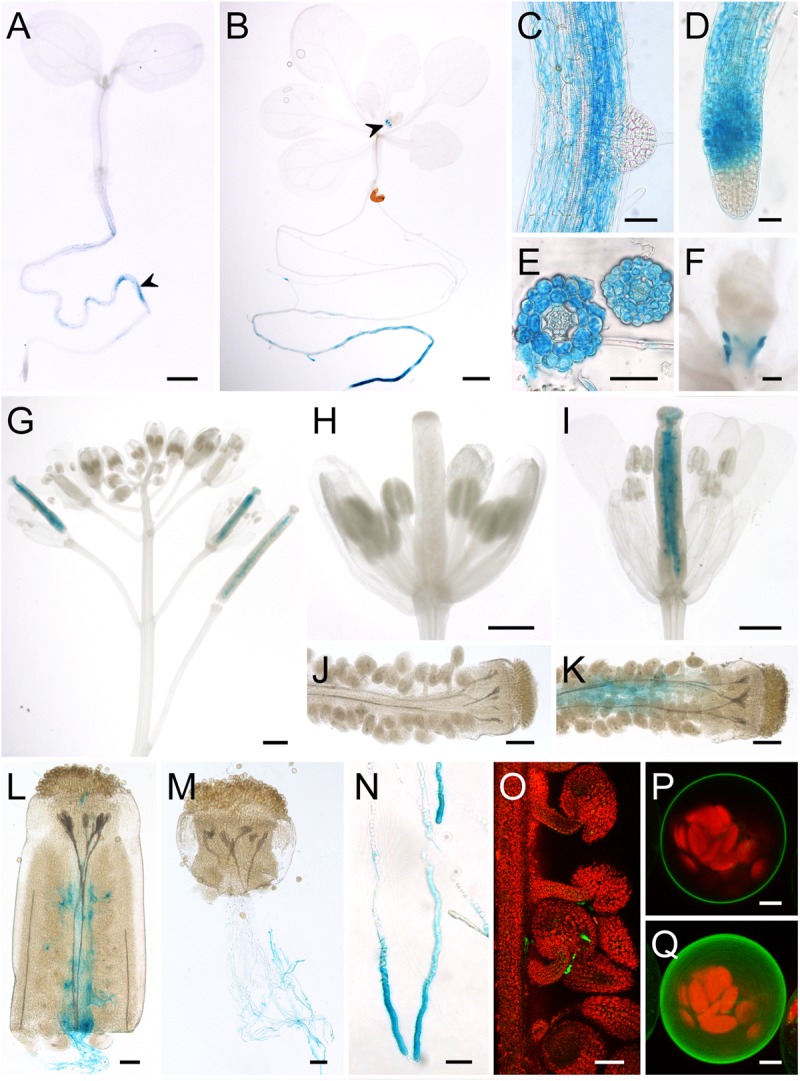
Analysis of p*AtSUC7:AtSUC7*g-reporter plants and subcellular localization of AtSUC7_Col-0_. **(A–N)** Histochemical detection of β-glucuronidase activity in Col-0 expressing p*AtSUC7:AtSUC7*g-*GUS*. **(A)** Five-day-old seedling with GUS staining in the main root (arrowhead). **(B)** Two-week-old seedling with GUS activity in roots and stipules (arrowhead). **(C)** Main root with lateral root primordium. **(D)** Tip of the main root at higher magnification. **(E)** Root cross sections in the differentiation (left) and elongation zone (right). **(F)** Stipules of a 2-week old seedling. **(G)** Inflorescence with flowers of different developmental stages. **(H)** Unpollinated flower. **(I)** Pollinated flower. **(J,K)** Peeled ovary of unpollinated **(J)** or pollinated **(K)** flower. **(L,M)** Pollen tubes grown semi-*in vivo* through a WT stigma with **(L)** or without **(M)** a part of the ovary. **(N)** Pollen tubes grown semi-*in vivo* at higher magnification. **(O)** GFP fluorescence (green) of pollen tubes in a peeled ovary of a p*AtSUC7:AtSUC7*g-*GFP* reporter plant. Chlorophyll autofluorescence is given in red. **(P,Q)** Single optical section **(P)** and maximum projection **(Q)** of an Arabidopsis mesophyll protoplast expressing *GFP-AtSUC7* under the control of the *35S* promoter. Scale bars: 1 mm in **(A,B,D)**; 50 μm in **(C,D,E,L,M,O)**; 100 μm in **(F,J,K)**; 500 μm in **(G–I)**; 20 μm in **(N)**; 5 μm in **(P,Q)**.

*AtSUC7*_Col-0_ reporter plants were obtained by transformation of Col-0 plants with the vectors pTR3 (GUS) and pTR4 (GFP) containing a 1,896-bp fragment of the *AtSUC7*_Col-0_ promoter, the complete genomic sequence of *AtSUC7*_Col-0_ and the respective reporter gene sequence. During plant vegetative growth *AtSUC7-GUS* expression became first visible in the roots of 5-day-old seedlings (**Figure 3A**). In 2-week-old seedlings GUS staining was more intense in the distal parts of main and lateral roots (**Figure 3B**) but the tips of lateral (**Figure 3C**) and main roots (**Figure 3D**) themselves showed no blue staining. Cross sections of stained roots revealed that the staining originated from all tissues of the root near the root tip, but was missing in the stele of the differentiation zone (**Figure 3E**). The only other vegetative tissue showing GUS activity were the stipules (**Figures 3B,F**). In flowers, GUS staining was detected in pollinated pistils only (**Figures 3G,I**). The blue staining in pistils originated from the transmitting tract (**Figure 3K**) and was missing in unpollinated flowers (**Figures 3H,J**). Cross-pollination of WT stigmata with pollen of *AtSUC7*g:*GUS* expressing plants in a semi-*in vivo* assay resulted in strong blue staining of pollen tubes (**Figures 3L,M**). This showed that the blue staining in the transmitting tract was caused by *AtSUC7:GUS* expression in pollen tubes. Interestingly, in semi-*in vivo* experiments GUS staining of pollen tubes was more intense when they grew through a longer section of the pistil (**Figure 3L**) and detailed imaging of pollen tubes revealed that the GUS activity was highest in the distal parts of the pollen tubes (**Figure 3N**). Together with the fact that no GUS staining was observed in pollen grains in anthers these results indicate that *AtSUC7*_Col-0_ expression is induced during pollen tube growth through the pistil. The restriction of *AtSUC7*_Col-0_ expression to the male gametophyte could be confirmed by analyses of plants transformed with the p*AtSUC7*:*AtSUC7*g-*GFP* construct. In pollinated pistils GFP fluorescence originated from pollen tubes growing toward the ovules, but could not be seen in any other tissue (**Figure 3O**).

Interestingly, reporter plants expressing *GUS* directly under the control of the *AtSUC7*_Col-0_ promoter without the genomic sequence of *AtSUC7*_Col-0_ (p*AtSUC7*:*GUS*) showed GUS staining not only in pollen tubes and roots. GUS activity in those plants was detected in roots and leaves of seedlings, especially in stomata and trichomes, as well as in flower stalks, sepals, ovaries, filaments, and pollen tubes (Supplementary Figure 1). This indicates that the genomic sequence of *AtSUC7* contains elements that regulate the expression of the gene and restrict its expression to pollen tubes and roots.

The subcellular localization of AtSUC7_Col-0_ was analyzed by expression of *AtSUC7c-GFP* and *GFP-AtSUC7c* fusion constructs in Arabidopsis mesophyll protoplasts under the control of the *35S* promoter. In constructs with AtSUC7c_Col-0_ directly attached to GFP, the fusion proteins all remained in the endomembrane system (Supplementary Figure 2). Therefore, *AtSUC7*c_Col-0_ was inserted by LR reactions into pMDC43 and pMDC83 (Curtis and Grossniklaus, 2003) yielding expression plasmids that coded for a spacer of at least 18 amino acids between GFP and AtSUC7_Col-0_. The AtSUC7-spacer-GFP fusion proteins still labeled the endomembrane system (Supplementary Figure 2), but the GFP-spacer-AtSUC7 fusions clearly localized to the plasma membrane (**Figures 3P,Q**). This indicates that AtSUC7_Col-0_ is a plasma membrane protein like AtSUC6_Col-0_ and that the endomembrane localization of the other constructs was caused by interference of the GFP fusion with protein targeting.

### Functional Characterization of *AtSUC6*_Col-0_ and *AtSUC7*_Col-0_ by Heterologous Expression in Yeast

**FIGURE 4:**
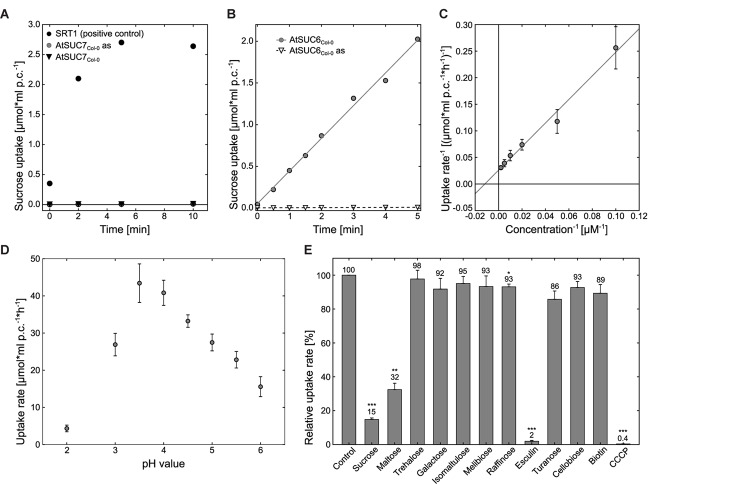
Analysis of AtSUC7_Col-0_ and AtSUC6_Col-0_ transport properties in transgenic baker’s yeast. **(A)** Uptake analysis of ^14^C-sucrose into yeast strains TRY1002 (black triangles) or TRY1001 (gray circles) expressing *AtSUC7c*_Col-0_ in sense or antisense (as) orientation, respectively, per ml packed cells (p.c.) at an initial outside concentration of 100 μM sucrose at pH 5.5. Yeast strain SEY2102 expressing the sucrose transporter *Srt1* was used as a positive control for sucrose uptake (black circles). **(B)** Uptake of ^14^C-sucrose into AtSUC6_Col-0_ strain TRY1039 (circles) and AtSUC6_Col-0_-antisense (as) control strain TRY1040 (triangles) per ml packed cells (p.c.) at an initial outside concentration of 100 μM sucrose at pH 5.5. **(C)** For the calculation of the *K*_M_ value of AtSUC6_Col-0_ for sucrose uptake according to Lineweaver–Burk uptake rates of TRY1039 for increasing concentrations of ^14^C-sucrose were determined. The plot represents mean values ± standard deviations of three biological replicates for each sucrose concentration. **(D)** Uptake rates of AtSUC6_Col-0_ for ^14^C-sucrose at different pH values at an initial outside concentration of 100 μM sucrose. **(E)** Analysis of AtSUC6_Col-0_ substrate specificity and sensitivity to uncouplers. Binding capacity of AtSUC6_Col-0_ for different sugars was determined by competitive inhibition of ^14^C-sucrose uptake (100 μM initial outside concentration) in the presence of non-radioactive sugars in 10-fold excess at pH 5.5. Addition of 1-mM cold sucrose was used as a control. CCCP was added to a final concentration of 50 μM. Means ± standard errors (SEs) of three independent biological replicates are shown. ^∗^*p* ≤ 0.05, ^∗∗^*p* ≤ 0.01, ^∗∗∗^*p* ≤ 0.001 by Student’s *t*-test.

To analyze whether the protein encoded by *AtSUC6*_Col-0_ represents a functional transport protein the CDS of *AtSUC6*_Col-0_ was amplified by PCR and expressed in the hexose- and invertase deficient *S. cerevisiae* strain CSY4000 (Rottmann et al., 2016). The forward primer was designed to attach 15 bp of the *STP1* 5′UTR in front of the start codon as this sequence has been reported to optimize the expression of plant genes in baker’s yeast (Stadler et al., 1995). As shown in **Figure 4B** yeast cells expressing *AtSUC6*c_Col-0_ in sense orientation (TRY1039) were able to take up ^14^C-sucrose, whereas yeast cells transformed with the *AtSUC6*c_Col-0_ *antisense* construct (TRY1040) did not accumulate radioactivity. TRY1039 cells were used for further studies of the transport properties of AtSUC6_Col-0_. The *K*_M_ value of AtSUC6_Col-0_ for sucrose was shown to be 81.2 ± 2.7 μM (**Figure 4C**), which is comparable to the *K*_M_ values of the closely related transporters AtSUC9 and AtSUC8. The maximum uptake rate *V*_max_ was 36.9 ± 8.8 μmol^∗^h^-1∗^ml^-1^ which is also comparable to that of other SUCs. *K*_M_ and *V*_max_ were determined at an external pH value of 5.5 which approximately resembles the physiological extracellular pH of most plant organs, but uptake measurements at different pH values revealed that the pH optimum for sucrose uptake via AtSUC6_Col-0_ is at pH 3.5 (**Figure 4D**). To test the possible uptake of other substances, transport of ^14^C-labeled sucrose was analyzed in the presence of various other substrates in 10-fold excess (**Figure 4E**). In these experiments uptake of sucrose was not reduced significantly upon addition of non-radioactive galactose, trehalose, isomaltulose, melibiose, turanose, or cellobiose, indicating that these mono- and disaccharides are not accepted by AtSUC6_Col-0_. The trisaccharide raffinose slightly interfered with sucrose uptake, indicating that the AtSUC6_Col-0_ binding pocket might eventually have a low affinity to this substrate. When maltose was added in 10-fold excess, uptake of ^14^C-sucrose was reduced to 32%, indicating that maltose might be an additional substrate of AtSUC6_Col-0_ as it is for all other SUCs analyzed so far. The inhibitory effect of maltose is similar to the inhibitory effect of non-radioactive sucrose, which points to the fact that AtSUC6_Col-0_ might transport both disaccharides at similar rates. Biotin, which has been described as an additional substrate of some SUCs, did not influence the sucrose transport activity of AtSUC6_Col-0_, suggesting that in contrast to many other SUCs, AtSUC6_Col-0_ does not transport biotin (**Figure 4E**). AtSUC6_Col-0_ showed the typical sensitivity of plant sucrose transporters to carbonyl cyanide *m*-chlorophenyl hydrazone (CCCP), an uncoupler of transmembrane proton gradients, indicating that AtSUC6_Col-0_ uses the energy of the proton gradient across the plasma membrane and works as a H^+^/sucrose symporter as it has already been shown for other SUCs (Sauer and Stolz, 1994; Ludwig et al., 2000; Meyer et al., 2000; Sauer et al., 2004; Schneider S. et al., 2012).

In summary, these results indicate that AtSUC6_Col-0_ is a high affinity, energy-dependent sucrose/H^+^-symporter. In contrast, expression of *AtSUC7*_Col-0_ did not enable the resulting yeast strain TRY1002 to take up radioactive ^14^C-sucrose (**Figure 4A**).

### Analysis of Sucrose Transporter Activities in Arabidopsis Protoplasts

**FIGURE 5:**
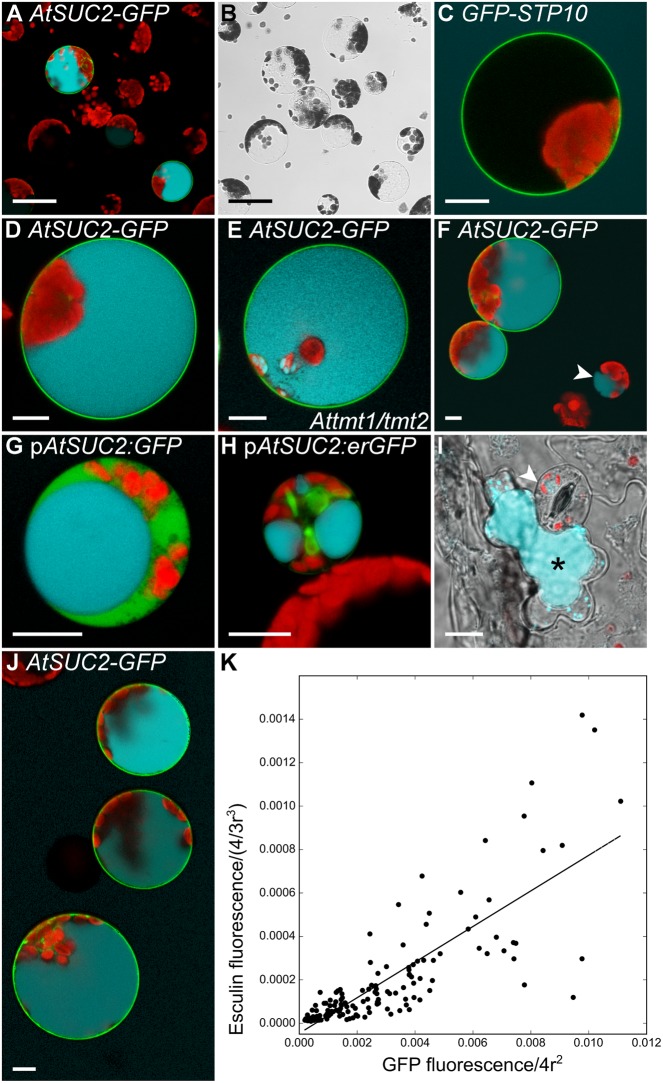
Control experiments for the establishment of a protoplast esculin assay. **(A–H,J)** Protoplasts expressing different *GFP*-fusion constructs were incubated with 1 mM esculin in W5 buffer (pH5.6) for 40 min. GFP is given in green, esculin fluorescence in cyan and chlorophyll autofluorescence in red. **(A)** Overview image of Col-0 protoplasts transformed with p*35S:AtSUC2*c-*GFP*. **(B)** Bright field to **(A)**. **(C)** Col-0 protoplast expressing p*35S:GFP-STP10*c. **(D)** Individual protoplast expressing p*35S:AtSUC2*c-*GFP* with esculin in the vacuole at higher magnification. **(E)** Protoplast of a *Attmt1/tmt2* knockout-plant transformed with p*35S:AtSUC2*c-*GFP*. **(F)** Col-0 protoplasts with p*35S:AtSUC2*c-*GFP*. The arrowhead indicates a small non-transformed protoplast showing esculin uptake. **(G)** Companion cell protoplast of a p*EPS1* line (stably transformed with p*AtSUC2:GFP*), labeled by cytosolic GFP. **(H)** GFP-labeled companion cell protoplast of p*MH5a* (stably transformed with a p*AtSUC2:erGFP* construct). **(I)** Leaf epidermal peel of Col-0 incubated with 1 mM esculin in W5 for 1 h. Overlay of bright field, esculin and chlorophyll fluorescence. The arrowhead points to a guard cell, the asterisk marks a neighboring subsidiary cell with esculin fluorescence. **(J)** WT protoplasts expressing p*35S:AtSUC2*c-*GFP* at different levels. **(K)** Correlation of GFP fluorescence in the plasma membrane and esculin fluorescence intensity in the vacuole of protoplasts transformed with p*35S:AtSUC2*c-*GFP*. Fluorescence intensities were normalized to the radius of the respective protoplast under the assumption of a spherical shape (*n* = 115). Scale bars: 50 μm in **(A,B)**, 10 μm in **(C–J)**.

Incorrect targeting of the foreign protein could be a reason for the missing sucrose uptake activity of *AtSUC7*_Col-0_ expressing yeast cells. Indeed, expression of *AtSUC7*_Col-0_*-GFP* and *GFP-AtSUC7*_Col-0_ constructs in yeast revealed that the resulting fusion proteins were not localized in the yeast plasma membrane (Supplementary Figures 2A–D). Possibly, also the AtSUC7_Col-0_ proteins without fluorophores were not correctly targeted to the yeast plasma membrane offering an explanation for the missing sucrose uptake. Another commonly used heterologous system for uptake measurements are *Xenopus* oocytes, but no sucrose uptake could be measured in oocytes expressing *AtSUC7*_Col-0_. However, AtSUC7_Col-0_ fusion constructs with GFP did not reach the plasma membrane also in this system (Supplementary Figures 2E,F). Gora et al. (2012) developed an assay to indirectly measure the sucrose uptake activity of transport proteins in yeast cells via the fluorescent sucrose analog esculin. As Arabidopsis mesophyll protoplasts were the only expression system where AtSUC7_Col-0_ was correctly targeted to the plasma membrane, the esculin uptake assay from yeast cells was transferred to protoplasts. To establish this new assay, the coding sequence of AtSUC2 for which esculin uptake activity has already been shown in the yeast assay (Gora et al., 2012) was transiently expressed in protoplasts as a fusion to *GFP*. Protoplasts transformed with *GFP-STP10*c served as a negative control as monosaccharide transporters do not transport esculin. Approximately 24 h after transformation protoplasts were incubated with 1-mM esculin for 40 min at pH 5.6. Subsequent confocal analyses of GFP and esculin fluorescences revealed that all protoplasts labeled by AtSUC2-GFP had taken up esculin (**Figures 5A,B**), whereas almost all other protoplasts showed no esculin-derived fluorescence. Protoplasts expressing *GFP-STP10* also showed no esculin uptake, even though the fusion protein clearly labeled the plasma membrane (**Figure 5C**). This confirmed that esculin uptake in the AtSUC2-GFP protoplasts was indeed mediated by AtSUC2 and was not a result of the transformation procedure or the synthesis of any additional plasma membrane protein. Detailed imaging of AtSUC2-GFP protoplasts that had taken up esculin revealed that the fluorescent molecule accumulated in the vacuole (**Figure 5D**). The sugar transporter proteins AtTMT1 and AtTMT2 have been reported to load sucrose into the vacuole (Schulz et al., 2011), indicating that they might also be responsible for the transport of esculin into the vacuole. However, when protoplasts of *Attmt1/tmt2* double-knock-out mutants (Wormit et al., 2006) were transformed with *AtSUC2-GFP* the cells were still able to accumulate esculin within the vacuole (**Figure 5E**), indicating that AtTMT1 and AtTMT2 are not or at least not exclusively responsible for esculin transport across the tonoplast.

Detailed statistical analyses of protoplast suspensions showed that also 2.2–6.7% of non-transformed protoplasts catalyzed esculin uptake (**Figure 5F**) in five individual experiments (*n* > 1,500 in total). The fact that those cells were in most cases rather small led to the hypothesis that these protoplasts might be derived from companion cells, the cell type known to express *AtSUC2* (Stadler and Sauer, 1996). When plants expressing cDNAs for free GFP or ER-bound GFP under the control of a 900-bp *AtSUC2* promoter (Imlau et al., 1999; Stadler et al., 2005b) were used for protoplast isolation, companion cell protoplasts were labeled with GFP (**Figures 5G,H**). Indeed, those labeled cells were able to accumulate esculin without further transformation with an additional sucrose transporter gene (**Figures 5G,H**). However, besides the labeled companion cells there were also some unlabeled cells that showed blue esculin fluorescence. Since these cells contained only few chloroplasts, it was analyzed, whether these cells are epidermal cells. To this end, epidermal peels of Col-0 leaves were incubated with 1-mM esculin. Subsequent microscopic analysis showed that the subsidiary cells of the stomatal complex were the only epidermal cells that accumulated esculin in young leaves (<5 mm) as used for protoplast isolation (**Figure 5I**). Interestingly, the ability of different epidermal cells to accumulate esculin changed during leaf development. In older leaves (>1.5 cm), subsidiary cells showed no fluorescence, but guard cells accumulated esculin (Supplementary Figure 3). Medium-sized leaves showed both, guard cells and subsidiary cells, taking up esculin (Supplementary Figure 3).

However, as companion cell and epidermis cell protoplasts can be distinguished easily from mesophyll cell protoplasts by their small size or their low number of chloroplasts, endogenous esculin uptake activity of these cells types does not interfere with the analysis of esculin uptake into transiently transformed protoplasts. The protoplast esculin assay even allows a relative quantification of esculin uptake as there is a near linear correlation between SUC-GFP fluorescence in the membrane and esculin fluorescence in the vacuole (**Figure 5J**), that can be analyzed by measuring the respective fluorescence intensities (**Figure 5K**).

**FIGURE 6:**
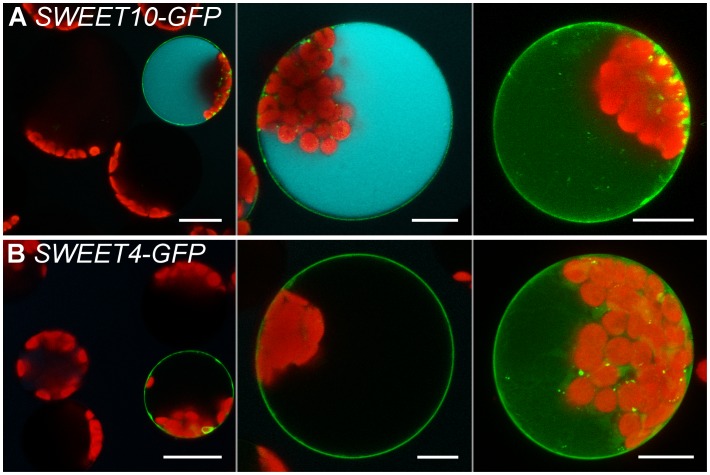
Esculin protoplast assay for SWEET transporters. Confocal images of protoplasts transformed with p*35S:SWEET10*c-*GFP* **(A)** or p*35S:SWEET4*c-*GFP* **(B)** and incubated with 1 mM esculin in W5 buffer (pH5.6) for 40 min. Left to right: overview image, single section and maximum projection without esculin detection channel of individual protoplasts. GFP is given in green, esculin fluorescence in cyan and chlorophyll autofluorescence in red. Scale bars: 20 μm in overview images, else: 10 μm.

It was furthermore tested whether the protoplast esculin assay is also useful to study the uptake activity of sucrose transporters of the SWEET family by expression of already characterized *SWEETs* in protoplasts. Cells transformed with the coding sequence for the sucrose specific SWEET10 (Chen et al., 2012) fused to *GFP* accumulated esculin, whereas protoplasts expressing the respective fusion construct for the glucose-specific SWEET4 (Chen et al., 2010) were not able to take up esculin (**Figure 6**). This proves that the esculin assay can also be applied to easily distinguish between sucrose- and monosaccharide-specific SWEET transporters.

**FIGURE 7:**
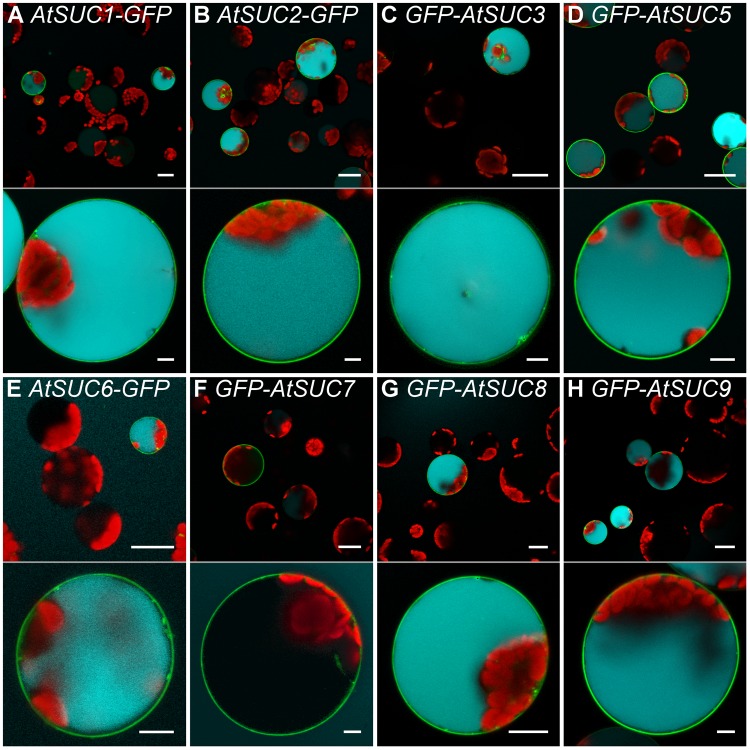
Esculin uptake into protoplasts via different AtSUCs. **(A–H)** Confocal images of Arabidopsis mesophyll protoplasts expressing *GFP* fusions of different *AtSUC*s under the control of the *35S* promoter as indicated. Protoplasts were incubated with 1-mM esculin for 40 min. Upper part: overview image. Bottom part: single optical sections of individual protoplasts. GFP is given green, esculin fluorescence in cyan and chlorophyll autofluorescence in red. Scale bars: 25 μm in overview images, 5 μm in single protoplast images.

**FIGURE 8:**
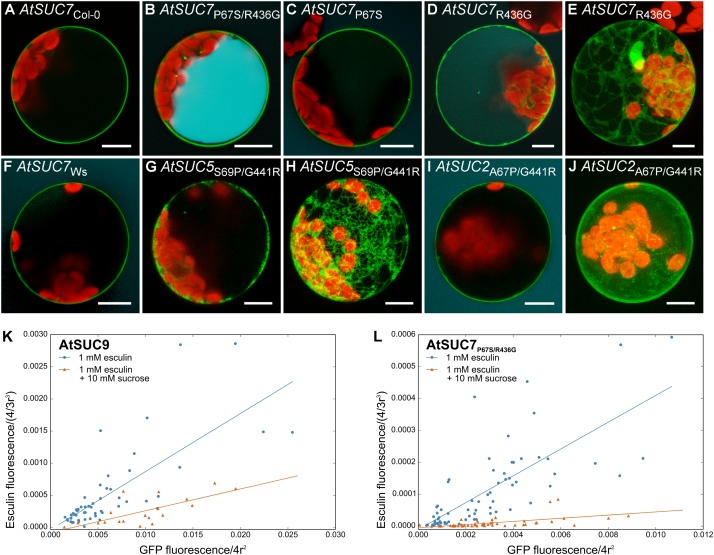
Protoplast esculin uptake assay for AtSUC sequence variants. **(A–J)** Confocal images of protoplasts transformed with *GFP* fusion constructs of *AtSUC7* wild type sequences of ecotypes Col-0 **(A)** or Ws **(F)**, or point mutated sequences of *AtSUC7*_Col-0_ **(B–E)**, *AtSUC5*_Col-0_ **(G,H)** or *AtSUC2*_Col-0_ **(I,J)** as indicated. GFP is given in green, esculin in cyan and chlorophyll autofluorescence in red. **(E,H,J)** Maximum projections without esculin detection channel. Scale bars: 10 μm. **(K,L)** Correlation of GFP fluorescence in the plasma membrane and esculin fluorescence intensity in the vacuole of protoplasts transformed with p*35S*:*GFP-AtSUC9* **(K)** or p*35S*:*GFP*-*AtSUC7*_P67S/R436G_ **(L)**. Protoplasts were incubated for 30 min with 1 mM esculin only (blue) or with 1 mM esculin in the presence of sucrose in 10-fold excess (orange). Fluorescence intensities were normalized to the radius of the respective protoplast under the assumption of a spherical shape (*n* > 19 protoplasts for each measurement).

Comparable to AtSUC2, transformation of protoplasts with the coding sequences for the type I sucrose transporters AtSUC1, AtSUC5, AtSUC6_Col-0_, AtSUC8, or AtSUC9 enabled the cells to accumulate esculin in their vacuoles (**Figure 7**). In yeast cells expressing *AtSUC6*_Col-0_ addition of esculin greatly interfered with sucrose uptake (**Figure 4E**), further confirming that esculin is an additional substrate of AtSUC6_Col-0_. The capacity of *AtSUC6*_Col-0_ and *AtSUC8* expressing protoplasts to take up esculin confirms that the respective proteins are mainly localized in the plasma membrane even though GFP fusion proteins are partially retained in the endomembrane system. Incomplete targeting to the plasma membrane was independent of the fusion of GFP to the *N*- or *C*-terminus for both proteins (Supplementary Figure 4). In contrast, targeting of AtSUC9 was dependent on the position of the GFP fusion. Whereas GFP-AtSUC9 localized to the plasma membrane, AtSUC9-GFP was mainly retained in the endoplasmatic reticulum (Supplementary Figure 4). However, some proteins still localized to the plasma membrane as the respective protoplasts were able to take up esculin (Supplementary Figure 4). Protoplasts with GFP-AtSUC3 were also able to accumulate esculin, which is surprising as AtSUC3 belongs to the group of type II SUCs that did not transport esculin in the yeast assay (Gora et al., 2012). In contrast to all other AtSUCs tested, the expression of *GFP-AtSUC7*_Col-0_ in protoplasts did not enable the cells to accumulate esculin even though the fusion proteins are clearly localized in the plasma membrane (**Figures 7F**, **8A**).

The alignment of the AtSUC7_Col-0_ amino acid sequence and all other AtSUCs (Supplementary Figure 5A) revealed two amino acids that are conserved in all AtSUCs except AtSUC7_Col-0_. The extracellular loop between TMD 1 and 2 contains a proline residue instead of a serine or an alanine and the cytoplasmic loop between TMD 10 and 11 carries an arginine, where all other AtSUCs display a glycine (Supplementary Figure 5A). Either one or both of the conserved amino acids were introduced at the respective positions of AtSUC7_Col-0_ by site directed mutagenesis leading to AtSUC7_P67S_, AtSUC7_R436G_, and AtSUC7_P67S/R436G_. The resulting fusion proteins with GFP were analyzed in protoplasts. The substitution R436G resulted in the partial accumulation of the GFP-AtSUC7_R436G_ fusion protein in the endoplasmatic reticulum (**Figures 8D,E**), but transformed cells seemed to accumulate some esculin (**Figure 8D**). In protoplasts with AtSUC7_Col-0_ carrying the substitution P67S the fusion protein was localized at the plasma membrane but the cells showed no esculin uptake (**Figure 8C**) similar to the WT AtSUC7_Col-0_ (**Figure 8A**). However, when both amino acids were substituted at the same time, protoplasts transformed with GFP-AtSUC7_P67S/R436G_ were able to take up esculin (**Figure 8B**). If esculin uptake indeed reflects a sucrose uptake activity, one would expect a reduction of esculin uptake in the presence of sucrose due to substrate competition. In control experiments with protoplasts expressing the functional sucrose carrier *AtSUC9* as a control we observed a severe reduction of esculin uptake in the presence of sucrose in 10-fold excess (**Figure 8K**). Similarly, esculin uptake via AtSUC7_P67S/R436G_ (**Figure 8L**) was reduced by excess sucrose indicating that the point mutations not only restored the esculin uptake activity of AtSUC7_Col-0_ but also its capability to transport sucrose. To test whether S67 or G436 are essential for AtSUC transporter function, the respective positions were exchanged in AtSUC5 and AtSUC2 to the amino acids of AtSUC7_Col-0_. Protoplasts transformed with *AtSUC5*_S69P/G441R_ (**Figures 8G,H**) showed that the mutation of the two amino acids interfered with plasma membrane localization of AtSUC5 (**Figure 7D**). In contrast AtSUC2_A67P/G441R_-GFP clearly localized to the plasma membrane (**Figures 8I,J**) but nevertheless the mutated protein did not mediate esculin uptake (**Figure 8I**), confirming the importance of the two amino acids for transporter function. The sequence of AtSUC7 in the ecotype Ws differs in eight amino acids from the Col-0 sequence (Supplementary Figure 5B), among them the two essential amino acids. Therefore, protoplasts expressing *AtSUC7*_Ws_ were tested for esculin uptake. As shown in **Figure 8F**, AtSUC7_Ws_ was not able to mediate esculin transport across the plasma membrane probably due to the six further amino acid substitutions between AtSUC7_Col-0_ and AtSUC7_Ws_ (Supplementary Figure 5B). In summary, the restoration of esculin uptake activity of AtSUC7_Col-0_ by the exchange of only two amino acids indicates that the failure to measure a transport activity of WT AtSUC7_Col-0_ was not due to experimental conditions like fusion to GFP. In contrast, it seems more likely that AtSUC7_Col-0_ is no functional transporter for esculin. However, it cannot be excluded that AtSUC7_Col-0_ might transport sucrose or another substrate.

### Characterization of *Atsuc6* T-DNA Insertion Lines

**FIGURE 9:**
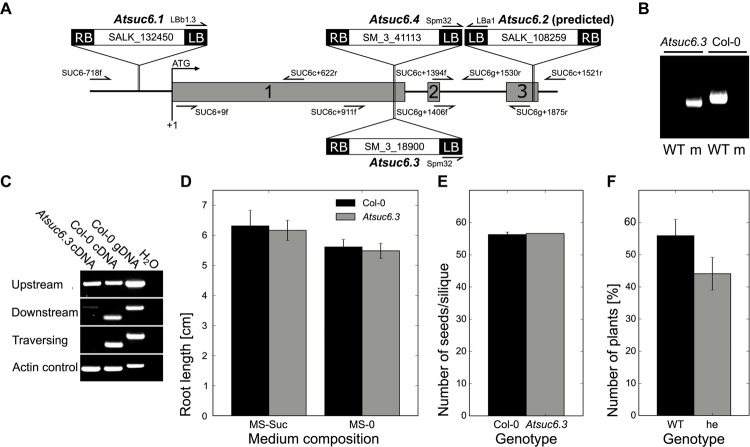
Identification and characterization of *Atsuc6* T-DNA insertion lines. **(A)** Genomic organization of *AtSUC6*. Introns and untranslated regions are shown as black lines; exon regions containing coding sequences are represented by numbered gray bars. Arrows indicate the primers used for PCRs shown in **(B,C)**. The positions of the T-DNA insertions *Atsuc6.1*–*Atsuc6.4* are marked. LB, left border; RB, right border. **(B)** PCR products obtained from genomic DNA preparations of homozygous *Atsuc6.3* and WT plants with primer combinations for the detection of wild type (WT) and the mutant allele (m). For primer combinations see Supplementary Table 2. **(C)** RT-PCR analyses of RNA obtained from flowers of a homozygous *Atsuc6.3* mutant plant and a WT plant with primers amplifying either the *AtSUC6* sequence traversing, upstream of or downstream of the insertion (Supplementary Table 3). WT genomic DNA was used as control for contaminations with genomic DNA. **(D)** Length of main roots of 14-day-old *Atsuc6.3* and WT seedlings on MS-0 or MS medium supplemented with 2% (w/v) sucrose. Means of three biological replicates ± SD are shown. *n* > 30 for each genotype. **(E)** Average number of seeds/silique ± SD of *Atsuc6.3* and WT plants after self-pollination. *n* > 50 siliques/genotype. **(F)** Genotypes of F1 descendants of a cross-pollination experiment with heterozygous *Atsuc6.3*/*AtSUC6* pollen and pistils from a WT plant. Bars represent mean values (±SE) of WT and heterozygous plants in the F1 generation resulting from eight independent crossings (*n* = 76 in total).

To investigate the potential physiological role of AtSUC6_Col-0_, four T-DNA lines with a predicted insertion in the respective gene were analyzed. Plants homozygous for the insertions *Atsuc6.3* (SM_1.8900) and *Atsuc6.4* (SM_3.41113) were identified by PCR (**Figure 9B**). Sequencing of the mutant alleles revealed that both insertions are at the same position 1,184 bp after the start codon of *AtSUC6*_Col-0_ (**Figure 9A**). The complete loss of *AtSUC6*_Col-0_ full-length transcripts was confirmed for *Atsuc6.3* by comparative RT-PCR analyses of flower-derived total RNA from homozygous mutants and WT plants (**Figure 9C**). No remaining *AtSUC6*_Col-0_ transcripts could be detected down-stream of the insertion, but truncated mRNAs from the regions upstream of the insertions could still be amplified from *Atsuc6.3*. However, a potential translation of these mRNA fragments would lead to truncated and therefore non-functional AtSUC6 proteins lacking the last three transmembrane domains. Homozygous plants were also identified for *Atsuc6.1* (SALK_132450), but the insertion in the promoter region led to an upregulation of *AtSUC6*_Col-0_ expression and no T-DNA insertion could be detected at the predicted site in *Atsuc6.2* (SALK_108259). Therefore, *Atsuc6.3* was used for all further analyses. As p*AtSUC6:AtSUC6*g-*GUS* reporter plants indicated a strong expression of *AtSUC6*_Col-0_ in the vascular tissue of roots and leaves of seedlings, plant and root development of *Atsuc6.3* was analyzed. However, *suc6.3* plants developed normally both on potting soil and on MS plates and no differences in root length on medium with or without sucrose could be observed in comparison to WT plants (**Figure 9D**). Even though *AtSUC6*_Col-0_ is expressed in both, the male and the female gametophyte (synergid cells), *Atsuc6.3* plants were self-fertile and produced viable seeds in the same quantity as WT plants (**Figure 9E**). To directly compare the fertility of WT and *Atsuc6.3* mutant pollen, a cross-pollination assay was performed by pollinating WT pistils with pollen of heterozygous *Atsuc6.3*/*AtSUC6* plants. The approximate 50:50 segregation ratio of WT to heterozygous plants in the descendant generation (**Figure 9F**) showed that the *Atsuc6.3* and the WT allele were inherited equally, indicating that the loss of AtSUC6_Col-0_ did not interfere with pollen tube function.

### Characterization of *Atsuc7* T-DNA Insertion Lines

**FIGURE 10:**
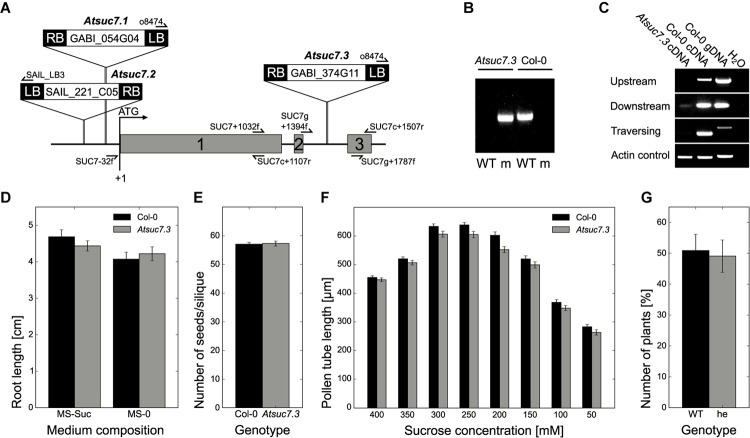
Identification and characterization of *Atsuc7* T-DNA insertion lines. **(A)** Genomic organization of *AtSUC7*. Introns and untranslated regions are shown as black lines; exon regions containing coding sequences are represented by numbered gray bars. Arrows indicate the primers used for PCRs shown in **(B,C)**. The positions of the insertion in the T-DNA lines are marked. LB, left border; RB, right border. **(B)** PCR products obtained from genomic DNA preparations of homozygous *Atsuc7.3* and WT plants. Primer combinations for the detection of wild type (WT) and mutant allele (m) are listed in Supplementary Table 2. **(C)** RT-PCR analyses of pollen tube RNA obtained from a homozygous *Atsuc7.3* mutant and a WT plant with primers (Supplementary Table 3) amplifying either the *AtSUC7* sequence traversing, upstream of or downstream of the insertion. WT genomic DNA was used a control for genomic contaminations. **(D)** Length of main roots of 12-day-old *Atsuc7.3* and WT seedlings on MS-0 or MS medium supplemented with 2% (w/v) sucrose. Means of five biological replicates ± SE are shown. *n* > 50 for each genotype. **(E)** Average number of seeds/silique ± SD of *Atsuc7.3* and WT plants after self-pollination. *n* > 55 siliques/genotype. **(F)** Pollen tube lengths of WT and *Atsuc7.3 in vitro.* Pollen were grown on medium with different sucrose concentrations for 8 h. Bars represent mean values of three biological replicates ± SE (*n* > 500 in total per sucrose concentration for each genotype). **(G)** Genotypes of F1 descendants of a cross-pollination experiment with heterozygous *Atsuc7.3*/*AtSUC7* pollen and pistils from a WT plant. Bars represent mean values (±SE) of WT and heterozygous plants in the F1 generation resulting from seven independent crossings (*n* = 89 in total).

Uptake measurements with esculin indicated that AtSUC7_Col-0_ is probably no functional transport protein. To test, whether the AtSUC7_Col-0_ protein might have another physiological function in plants for example as sucrose sensor or regulator of other SUCs, T-DNA insertion lines were characterized. For three T-DNA insertion lines homozygous plants could be identified by PCR (**Figure 10B**). However, the insertions of *Atsuc7.1* (GABI_054G04) and *Atsuc7.2* (SAIL _221_C05) lie in the promoter region (**Figure 10A**) and lead to an upregulation instead of a knockout of *AtSUC7*_Col-0_. *Atsuc7.3* (GABI_374G11) carries an insertion in the second intron 1,595 bp downstream of the start ATG (**Figure 10A**). RT-PCR analysis of pollen tube derived mRNA from homozygous *Atsuc7.3* in comparison to WT mRNA confirmed the absence of full-length *AtSUC7*_Col-0_ transcripts in this line. As also no truncated upstream or downstream fragments could be amplified from *Atsuc7.3* RNA preparations, this indicates that *Atsuc7.3* is a real *AtSUC7*_Col-0_ knockout line (**Figure 10C**). *Atsuc7.3* plants were analyzed regarding the lengths of roots and pollen tubes as these are the *AtSUC7*_Col-0_ expression sites identified by p*AtSUC7*:*AtSUC7*g reporter plants. On MS medium with or without sucrose root length of *Atsuc7.3* plants was not altered compared to WT plants (**Figure 10D**). Self-fertilized homozygous plants produced normal amounts of seeds and pollen tube growth was not altered compared to WT *in vitro* (**Figures 10E,F**). A 50:50 segregation ratio of WT to heterozygous plants in the descendants of a cross-pollination assay with *Atsuc7.3*/*AtSUC7* pollen on WT stigmata showed that pollen tube function *in vivo* is not affected in *Atsuc7.3* (**Figure 10G**).

## Discussion

This paper presents a detailed characterization of the putative sucrose transporter genes *AtSUC6* and *AtSUC7* in Arabidopsis ecotype Col-0, both of which had been described as pseudogenes in other ecotypes (Sauer et al., 2004). Expression of the *AtSUC6*_Col-0_ coding sequence in baker’s yeast and in protoplasts revealed that AtSUC6_Col-0_ is a plasma membrane-localized high-affinity H^+^/sucrose symporter. AtSUC6_Col-0_ shares these features all other SUCs described so far (Sauer, 2007; Kühn and Grof, 2010) with the exception of AtSUC4 which is localized in the tonoplast (Schneider S. et al., 2012). Competitive inhibition experiments indicated that AtSUC6_Col-0_ accepts also maltose and small amounts of raffinose as substrates. Maltose is a classical substrate of all plant SUCs described so far (Sauer, 2007; Kühn and Grof, 2010), whereas uptake of raffinose, which is a compound of the Arabidopsis phloem sap (Haritatos et al., 2000) has only been described for PmSUC2 (Gahrtz et al., 1994). It has been discussed that biotin transport may be a common feature of plant SUCs (Ludwig et al., 2000), but its uptake is not mediated by AtSUC6_Col-0_. The *K*_M_ value of 81 μm of AtSUC6_Col-0_ for sucrose is the lowest value determined for a plant SUC in yeast so far. The closely related AtSUC8 also has a high affinity (*K*_M_ = 150 μm) (Sauer et al., 2004), whereas the *K*_M_ values of other SUCs range from 0.45 to 13.7 mM (Sauer and Stolz, 1994; Kühn, 2003; Doidy et al., 2012). Only for AtSUC9 a lower *K*_M_ value (66 μm) has been determined in *Xenopus* oocytes (Sivitz et al., 2007). However, in the yeast system the *K*_M_ of this transporter was 500 μm (Sauer et al., 2004). The existence of a family of sucrose transporters with quite different *K*_M_ values is well in line with the various apoplastic sucrose concentrations in plants. Around guard cells the sucrose concentration is about 150 mM, in the apoplast of mesophyll cells it reaches 500 mM and near the phloem it even exceeds 500 mM (Giaquinta, 1983; Outlaw and De Vlieghere-He, 2001; Sivitz et al., 2007). In contrast to most SUCs with pH optima in the range of pH 5-6, AtSUC6_Col-0_ transports best at pH 3.5. pH optima around pH 3 have also been described for some SUCs of other species, for example BvSUT1 (Nieberl et al., 2017) or StSUT1 (Krügel et al., 2013). Even though the normal apoplastic pH lies between 5.3 and 6.7 (Gao et al., 2004), under certain conditions like auxin-dependent cell expansion it may reach pH values close to 4.0. As this acidification occurs via membrane integral H^+^-ATPases the pH value directly at the plasma membrane may even be lower than in the remaining cell wall (Krügel et al., 2013). It has even been discussed that the different pH optima of SUCs might represent a mode of transporter activity regulation by altering the extracellular pH value (Wippel et al., 2010).

Promoter-reporter gene analyses demonstrated *AtSUC6*_Col-0_ expression in the vasculature of leaves, in a confined region of root tips, in synergid cells and pollen tubes. The two cell files stained by p*AtSUC6:AtSUC6*g*-GUS* expression in root tips might represent cells of the early protophloem. Phloem unloading in the root tip has been reported to occur mainly via the symplastic route through plasmodesmata (Oparka et al., 1994; Stadler et al., 2005b). However, the coexistence of symplastic and apoplastic phloem loading in root tips has also been discussed. In maize, symplastic diffusion of sugars from the phloem cannot cover all the carbon requirements of the root meristem (Bret-Harte and Silk, 1994). As the cells of the young protophloem are still in a process of differentiation, the expression of a gene for an additional sucrose transporter like AtSUC6_Col-0_ might be useful to cover their increased energy demand. The same reason might explain the expression of *AtSUC6*_Col-0_ at the base of developing lateral roots. In addition to *AtSUC6*_Col-0_ also *AtSUC1*, *AtSUC2*, *AtSUC3*, and *AtSUC4* are expressed in roots. However, expression of *AtSUC1*, *AtSUC2*, or *AtSUC3* does not overlap with *AtSUC6*_Col-0_ expression as GUS staining in reporter lines for *AtSUC1* and *AtSUC2* ended more proximal compared to AtSUC6_Col-0_ (Truernit and Sauer, 1995; Sivitz et al., 2007) and *AtSUC3* expression is confined to the most distal parts of the root tip (Meyer et al., 2004). *AtSUC4* expression in the root tip is similar to the *AtSUC6*_Col-0_ pattern, but the AtSUC4 protein is located in the tonoplast (Schneider S. et al., 2012). However, *Atsuc6* mutants did not show any root phenotype. This indicates that uptake of sucrose via AtSUC6_Col-0_ either might not be essential for root growth, that AtSUC6_Col-0_ function might be complemented by upregulation of other SUCs or that cells can also be supplied by cleavage of sucrose via cell-wall invertases and subsequent uptake of monosaccharides via STPs as some of them also show a high expression in roots (Büttner, 2010; Rottmann et al., 2016, 2018). The expression of *AtSUC6*_Col-0_ in the vasculature of leaves is accompanied by the expression of *AtSUC2*, *AtSUC3*, *AtSUC4*, and *AtSUC9* in Arabidopsis (Stadler and Sauer, 1996; Meyer et al., 2004; Sivitz et al., 2007; Schneider S. et al., 2012). AtSUC2 is localized in companion cells (Stadler and Sauer, 1996) and is necessary and sufficient for phloem loading (Gottwald et al., 2000). AtSUC3 has been localized to sieve elements and probably parenchymatic cells and might be involved in the retrieval of sucrose lost from the phloem during transport (Meyer et al., 2000, 2004). The strong expression of *AtSUC6*_Col-0_ mainly in the major veins might indicate that also AtSUC6_Col-0_ functions in the retrieval of sucrose in the transport phloem. Its exceptional high affinity predestines AtSUC6_Col-0_ for the reuptake of sucrose at very low extracellular concentrations.

The highest expression of *AtSUC6*_Col-0_ was observed in the male and female gametophytes. In ovules of p*AtSUC6*:*AtSUC6*g-*GFP* reporter lines the fusion protein clearly labeled the synergid cells. Whereas ovule primordia are symplastically connected to the phloem, sugar transport within mature ovules has to pass several apoplastic steps (Stadler et al., 2005a; Werner et al., 2011). Symplastic gaps between the outer and the inner integument as well as the inner integument and the embryo sac are bypassed by sugar efflux via SWEETs and reuptake into the next symplastic domain through SUCs or STPs (Büttner, 2010; Chen et al., 2015; Rottmann et al., 2018). Finally, the cells of the embryo sac need to import the released sugars from the apoplast via transport proteins. The central cell can take up monosaccharides via STP8 (Rottmann et al., 2018) and is symplastically connected to the antipodal cells, but not to the synergids (Mansfield et al., 1991). Therefore, AtSUC6_Col-0_ might be involved in direct nutrient supply to the synergid cells which are important for pollen tube attraction and reception. The additional expression of *AtSUC1* (Feuerstein et al., 2010) and *AtSUC9* (Sivitz et al., 2007) in this cell type might explain why the knockout of *AtSUC6* does not interfere with plant fertility.

Similar to the embryo sac also pollen tubes are symplastically isolated. As their rapid tip-growth consumes a lot of metabolic energy it is likely that AtSUC6 supports the uptake of nutrients from the surrounding tissue. In addition to AtSUC6 the monosaccharide transporters STP4, STP6, STP8, STP9, STP10, and STP11 as well as the sucrose transporters AtSUC1, AtSUC3, AtSUC4, and AtSUC9 (Stadler et al., 1999; Meyer et al., 2004; Qin et al., 2009; Büttner, 2010; Leydon et al., 2013, 2014; Rottmann et al., 2018) have been detected in pollen tubes. The parallel expression of sucrose transporters, cell-wall invertases and monosaccharide transporters in pollen tubes points toward a high physiological redundancy and might offer an explanation for the missing pollen tube phenotype of *Atsuc6* mutants. Interestingly, in pollen tubes of p*AtSUC6:SUC6*g-*GUS* plants the fusion protein was only detected after pollen tubes grew through a part of the transmitting tract. This indicates that transcription or translation of *AtSUC6*_Col-0_ is induced after interaction of the pollen tube with the maternal tissue. A similar regulation has been described for *AtSUC7*, *AtSUC8*, and *AtSUC9.* Expression of the three genes was only induced when pollen tubes were grown semi-*in vivo* through the stigma but not when they were grown *in vitro* (Qin et al., 2009; Leydon et al., 2013). In contrast to these results, for *AtSUC6*_Col-0_ expression growth through the stigma was not sufficient, indicating a different induction mechanism for *AtSUC6*_Col-0_ that depends on factors only present in the transmitting tract. This late induction of the high affinity AtSUC6_Col-0_ might be a preparation for the exit of the pollen tube from the nutrient rich extracellular matrix of the transmitting tract.

Analyses of p*AtSUC7:AtSUC7*g reporter lines including the genomic region of *AtSUC7*_Col-0_ indicated that *AtSUC7*_Col-0_ is only expressed in sink tissues, namely roots and pollen tubes. Interestingly, the comparison with p*AtSUC7:GUS* lines showed that the restriction of *AtSUC7*_Col-0_ expression to sink tissues is mediated by intragenic regions, most likely the introns. The additional GUS staining observed in leaves, flower stalks, sepals, ovules and filaments of *pAtSUC7:GUS* plants suggests that the *AtSUC7*_Col-0_ promoter is active in more tissues than the protein is actually made in. A regulation by intragenic sequences has also been described for the sucrose transporter genes *AtSUC1*, *AtSUC9*, and *LeSUT1* as well as some other genes (Fiume et al., 2004; Rose, 2004; Sivitz et al., 2007; Weise et al., 2008). In contrast to *AtSUC7*_Col-0_ the inclusion of the genomic sequence of *AtSUC1*, *AtSUC9*, or *LeSUT1* into the reporter gene construct led to the expression of the respective gene in more tissues compared to the promoter-*GUS* fusions (Sivitz et al., 2007; Weise et al., 2008). Also in most other known examples, the presence of intragenic sequences expands the spatial or temporal expression of the genes (Fiume et al., 2004; Rose, 2004; Jeong et al., 2007). An intron-mediated restriction of gene expression to specific tissues as observed for *AtSUC7*_Col-0_ has only been described for *AGAMOUS* and *STP10* in Arabidopsis before (Sieburth and Meyerowitz, 1997; Rottmann et al., 2016).

Similar to *AtSUC6*_Col-0_, the expression of *AtSUC7*_Col-0_ increased during pollen tubes grew through the transmitting tract. Despite the precise regulation of *AtSUC7*_Col-0_ expression in roots and pollen tubes, *Atsuc7* knockout lines showed no differences compared to WT plants regarding pollen tube growth and fertility or root length. One explanation for this might be functional redundancy with other sugar transporters as described for AtSUC6. However, in an earlier publication *AtSUC7* had been described to be a pseudogene in other ecotypes on the base of uptake measurements in transgenic baker’s yeast (Sauer et al., 2004). Expression of GFP-labeled *AtSUC7*_Col-0_ in baker’s yeast or *Xenopus* oocytes at first indicated that mistargeting of AtSUC7_Col-0_ to internal membranes in those heterologous expression systems might be the reason why no sucrose uptake via AtSUC7_Col-0_ was detectable. Mistargeting may have been caused by misinterpretation of plant targeting signals in *Xenopus* and yeast. To circumvent the latter obstacle a novel assay for the analysis of SUC transporter activity was developed using Arabidopsis mesophyll protoplasts as expression system and the fluorescent sucrose analog esculin to monitor transporter activity.

We demonstrated that intact Col-0 mesophyll protoplasts did not take up esculin. Occasionally, contaminations with epidermal cell, guard cell or companion cell protoplasts represented a negligibly small and easy to identify subset of protoplasts that were able to accumulate esculin. The capability of companion cells to take up esculin was not surprising as they contain AtSUC2 in their plasma membranes to take up sucrose from the apoplast for phloem loading (Stadler and Sauer, 1996). That esculin is a substrate of AtSUC2 has been demonstrated before in *Xenopus* oocytes, in yeasts and by phloem loading with esculin after application to the leaf apoplasm (Gora et al., 2012; Reinders et al., 2012; Knoblauch et al., 2015). Mature guard cells have fewer and smaller chloroplasts and lower concentrations of Rubisco compared with mesophyll cells and, therefore, are unable to perform significant photosynthetic CO_2_ fixation (reviewed in Daloso et al., 2016). The resulting need for carbon supply via transporters is reflected in the expression of genes for the monosaccharide transporters STP1, STP4, and STP13 (Stadler et al., 2003; Leonhardt et al., 2004; Yang et al., 2008) and sucrose transporter AtSUC3 (Meyer et al., 2004). Interestingly, direct analysis of esculin uptake into cells of epidermal peels revealed that only mature guard cells of older leaves accumulated esculin whereas in younger leaves as used for protoplast isolation esculin accumulated in the subsidiary cells adjacent to young guard cells. This is well in line with the observation that immature guard cells are connected to adjacent cells via continuous plasmodesmata, which are sealed during the development of the mature cell wall (Wille and Lucas, 1984). This indicates that young guard cells might be provided with nutrients from adjacent cells. After this connection is closed during guard cell maturation they need sucrose transporters to take up sugars from the apoplasm. The lack of esculin fluorescence in young guard cells even though they are symplastically connected to the esculin accumulating subsidiary cells might be explained by the sequestration of esculin into the vacuoles of the subsidiary cells. Accumulation of esculin inside the vacuole was also observed in all further experiments and has been described in previous studies (Tattini et al., 2014; Knoblauch et al., 2015). However, the transporters mediating uptake of esculin across the tonoplast were not known. AtTMT1 and AtTMT2 are able to transport sucrose into the vacuole (Schulz et al., 2011), but the accumulation of esculin in the vacuoles of *Attmt1/tmt2* protoplasts showed that these transporters are at least not the only transporters mediating the observed uptake.

The lack of esculin fluorescence in mesophyll protoplasts and their accessibility to transient transformation made this cell type a convenient system to study the activity of sucrose transporters. Indeed, transformation of protoplasts with *AtSUC2-GFP* enabled the transformed cells which were identified by GFP fluorescence to take up esculin, whereas transformation with *STP10-GFP* did not. This indicates that esculin uptake was a specific result of *AtSUC2* expression. Also AtSUC1, AtSUC5, AtSUC6_Col-0_, AtSUC8, and AtSUC9 mediated esculin uptake which is consistent with previous reports that all SUCs of the phylogenetic type I-subgroup accept esculin as further substrate (Gora et al., 2012). Esculin uptake has also been shown for type I SUCs StSUT1 from potato (Gora et al., 2012) and BvSUT1 from sugar beet (Nieberl et al., 2017) in the yeast esculin assay. Interestingly, protoplasts expressing *AtSUC3-GFP* revealed that also type II SUCs are able to transport esculin, whereas previous studies in yeast indicated that esculin is no substrate of type II SUCs (Sivitz et al., 2007; Sun et al., 2010; Gora et al., 2012). A reason for this discrepancy might be the absence of protein modifications or interaction partners necessary for transporter function in the yeast system. Another explanation might be the use of the dicotyledonous AtSUC3 in this study whereas the report that type II SUCs don’t transport esculin was based on uptake analysis of type II SUCs from monocotyledons. Type II SUCs of monocotyledons form a separate subgroup within the type II clade (Peng et al., 2014) and they have been shown to have a generally narrower substrate specificity (Sivitz et al., 2007). Sucrose release from plant cells is usually mediated by the sucrose facilitators of the SWEET family, but it was not known so far if they also accept the sucrose analog esculin as a substrate. Transformation of protoplasts with *SWEET10* revealed that sucrose specific SWEETs (Chen et al., 2012) can mediate the uptake of esculin into protoplasts, whereas glucose specific SWEETs like SWEET4 (Chen et al., 2010) can’t. Even though SWEETs are passive facilitators (Chen et al., 2010, 2012), sucrose accumulated in *SWEET10* expressing protoplasts to a higher concentration than in the surrounding medium. The sequestration of esculin into the vacuole might keep the concentration in the cytosol low and thus enable further esculin uptake.

It is not clear whether esculin transport via sucrose transporters is of physiological relevance for plants or represents an artifact due to structural similarity. However, coumarins including esculin have been found in small quantities in Arabidopsis and they function in plant defense and iron acquisition (Kai et al., 2006; Schmid et al., 2014).

The expression of *AtSUC7*_Col-0_ in protoplasts did not enable the cells to take up esculin, but the point mutated variant AtSUC7_P67S/R436G_ mediated uptake. This indicates that the WT AtSUC7_Col-0_ is indeed a non-functional transporter for esculin and the missing transport activity is caused by the lack of two conserved amino acids. The importance of these amino acids for transporter function was underlined by the mutation of these amino acids in AtSUC2 which resulted in a non-functional transporter. The conserved serine/alanine residue at position 67 lies within the first extracellular loop. The modification of the adjacent histidine 65 in AtSUC1 led to changes in the transporter characteristics, indicating that the His-65 is involved in sucrose binding (Lu and Bush, 1998). Also in *Chlorella* glucose transporters HUP1 and HUP2, the first external loop was demonstrated to confer substrate specificity through a single acidic amino acid residue (Will et al., 1998). A structurally unrelated proline instead of serine in this region might therefore interfere with esculin binding and transport in AtSUC7_Col-0_. The additional amino acid exchanges between AtSUC7_Col-0_ and AtSUC7_Ws_ and the lacking esculin transport activity of AtSUC7_Ws_ even though this ecotype isoform contains the two conserved amino acids as well as the lack of phenotypes in *Atsuc7* mutant plants point toward random mutations and low functional relevance of the AtSUC7 protein. However, it cannot be excluded that the WT form of AtSUC7_Col-0_ might be able to transport another substrate or has some other regulatory function not related to its transport activity, for example as a part of a membrane complex, competitor, or sucrose sensor. The capability of sugar transporters to work additionally as sugar sensors has long been discussed as sucrose in plants seems to be sensed at the plasma membrane (Rolland et al., 2006) and sugar transporter homologs are involved in sugar sensing in yeasts (Özcan et al., 1998).

The analysis of AtSUC7 and the associated AtSUC point mutants in protoplasts demonstrated that the esculin protoplast assay is a useful tool to study sucrose transporters: As an *in planta* system it improves correct protein modification and targeting compared to the yeast system. The transient transformation of protoplasts allows fast results and the coexpression of several proteins. GFP-labeling of the protein of interest and the use of the fluorescent esculin as substrate enable the quantification of transport proteins in the membrane in comparison to substrate accumulation. The quantification of esculin uptake in the presence of other compounds allows the characterization of other possible substrates. It further provides the possibility to compare esculin uptake activity for example of point mutants, splice variants, transporters with and without interaction partners or phosphomimetic mutants.

## Author Contributions

RS conceived the research plans and supervised the experiments. TR performed most of the experiments. CFr generated the *AtSUC6* protoplast and yeast constructs. AL cloned the *AtSUC7*_Col-0_ entry vectors. SS provided plasmid pSS105. ND provided plasmids pND98, pND100, and pND101. CFi and PD constructed vector pGEM-GFP. PD provided assistance during the electrophysiological measurements. TR and RS designed the experiments and analyzed the data. TR wrote the article with contributions of RS, SS, and NS. NS discussed the data and supervised and complemented the writing.

## Conflict of Interest Statement

The authors declare that the research was conducted in the absence of any commercial or financial relationships that could be construed as a potential conflict of interest. The reviewer VZ and handling Editor declared their shared affiliation.

## Footnotes

1 http://arabidopsis.info/

2 http://gimp.org
