# Supplementary material for: Protoplast-Esculin Assay as a New Method to Assay Plant Sucrose Transporters: Characterization of AtSUC6 and AtSUC7 Sucrose Uptake Activity in Arabidopsis Col-0 Ecotype
Source: Front Plant Sci. 2018 Apr 23;9:430. doi: 10.3389/fpls.2018.00430 (PMC5925572; doi:10.3389/fpls.2018.00430)
Supplement: Supplementary file 1 [file Data_Sheet_1.pdf]

Protoplast-Esculin Assay as a New Method to Assay Plant Sucrose Transporters: Characterization of AtSUC6 and AtSUC7 Sucrose Uptake Activity in Arabidopsis Col-0 Ecotype

Theresa Rottmann, Carolin Fritz, Anja Lauter, Sabine Schneider, Cornelia Fischer, Nina Danzberger, Petra Dietrich, Norbert Sauer and Ruth Stadler

Supplementary Table 1: List of primers used for the detection of *AtSUC* transcripts in different tissues by RT-PCR.

Gene	Primer name	Primer sequence
AtSUC6	AtSUC6c+911f	5’-GTCGTGAGGTGTACGGTGGAGAC-3‘
	AtSUC6c+1521r	5’-CATGTATGGGTCCTTATCTAGTGCTACAATAA-3‘
AtSUC7	AtSUC7c+1032f	5’-TGGTATTGAAGGTATTAGTTAGGAAGATGGGA-3‘
	AtSUC7c+1507r	5’-GCATATATAATAAATAAAACGCATAAACTTTTTAAGG-3‘
ACTIN2	AtACT2g+846f	5’-ATTCAGATGCCCAGAAGTCTTGTT-3‘
	AtACT2g+1295r	5’-GAAACATTTTCTGTGAACGATTCCT-3‘

Supplementary Table 2: Primers used for PCR based genotyping of T-DNA insertion lines.

Mutant line	Amplified allele	Primer name	Primer sequence
Atsuc6.1	WT allele	AtSUC6-718f	5’-GAGGGAGACTGATTCGCCGG-3‘
		AtSUC6c+622r	5’-GCGCAATAGATGTCGCATGCTT-3‘
	mutant allele	SALK-LBb1.3	5’-ATTTTGCCGATTTCGGAAC-3‘
		AtSUC6c+622r	5’-GCGCAATAGATGTCGCATGCTT-3‘
Atsuc6.2	WT allele	AtSUC6g+1406f	5’-GCAGCTCCGGTGCCGG-3‘
		AtSUC6c+1521r	5’-CATGTATGGGTCCTTATCTAGTGCTACAATAA-3‘
	mutant allele	SALK-LBb1.3	5’-ATTTTGCCGATTTCGGAAC-3‘
		AtSUC6c+1521r	5’-CATGTATGGGTCCTTATCTAGTGCTACAATAA-3‘
Atsuc6.3&Atsuc6.4	WT allele	AtSUC6g+911f	5’-GTCGTGAGGTGTACGGTGGAGAC-3‘
		AtSUC6g+1530r	5’-CGTAAAGTAGAGATAATTGGAAACCGATCTC-3‘
	mutant allele	Spm32	5’-TACGAATAAGAGCGTCCATTTTAGAGTGA-3‘
		AtSUC6g+1530r	5’-CGTAAAGTAGAGATAATTGGAAACCGATCTC-3‘
Atsuc7.1	WT allele	AtSUC7-617f	5’-TTGATAACGTACAGCCAAGACATTTGATT-3‘
		AtSUC7-27r	5’-GGTCACTCATCTTCTTCACCCTTGTCTT-3‘
	mutant allele	GABI-Kat LB o8474	5’-ATAATAACGCTGCGGACATCTACATTTT-3‘
		AtSUC7-27r	5’-GGTCACTCATCTTCTTCACCCTTGTCTT-3‘
Atsuc7.2	WT allele	AtSUC7-617f	5’-TTGATAACGTACAGCCAAGACATTTGATT-3‘
		AtSUC7c+223r	5’-GACCGCAAAGCCAAATGAAAGATG-3‘
	mutant allele	AtSUC7-617f	5’-TTGATAACGTACAGCCAAGACATTTGATT-3‘
		SAIL_LB3	5’-TAGCATCTGAATTTCATAACCAATCTCGATACAC-3‘
Atsuc7.3	WT allele	AtSUC7g+1394f	5’-GGCGCCGGTCAAAGTATTTTTAAC-3‘
		AtSUC7c+1507r	5’-GCATATATAATAAATAAAACGCATAAACTTTTTAAGG-3‘
	mutant allele	GABI-Kat LB o8474	5’-ATAATAACGCTGCGGACATCTACATTTT-3‘
		AtSUC7c+1507r	5’-GCATATATAATAAATAAAACGCATAAACTTTTTAAGG-3‘

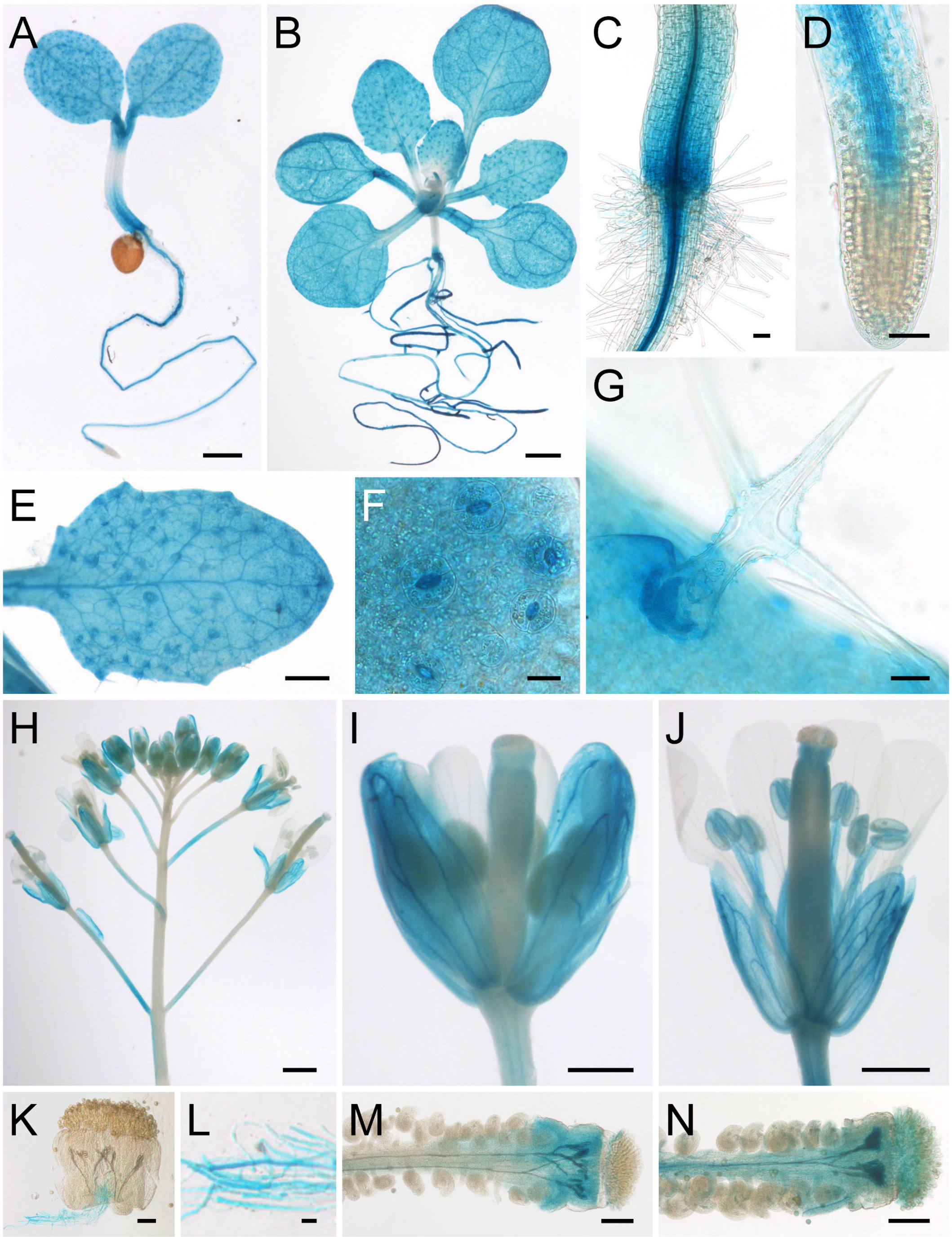

Supplementary Figure 1: **Analysis of *AtSUC7*$_{\mathbf{Col\text{-}0}}$ promoter activity by histochemical detection of ß-glucuronidase localization in p*AtSUC7:GUS* plants.** **(A)** 5-day-old seedling with GUS staining in roots, hypocotyl and cotyledons. **(B)** 2-week-old seedling. **(C)** Hypocotyl and proximal root end of 5-day-old seedling at higher maginfication showing GUS activity especially in the vascular tissue. **(D)** Lateral root with GUS staining absent from the tip. **(E)** Source leaf. **(F)** Epidermis with guard cells at higher magnification. **(G)** Trichome with strong GUS staining at the base. **(H)** Inflorescence with flowers of different developmental stages. **(I)** Unpollinated flower with GUS staining in the sepals. **(J)** Pollinated flower. **(K)** Pollen tubes grown semi-*in vivo* through a WT stigma. **(L)** Pollen tubes at higher magnification. **(M, N)** Peeled ovary of an unpollinated **(M)** or a pollinated **(N)** flower. Scale bars: 1 mm in A, B and H; 50 µm in C, D and K; 2.5 mm in E; 20 µm in F, G and L; 500 µm in I and J; 100 µm in M and N.

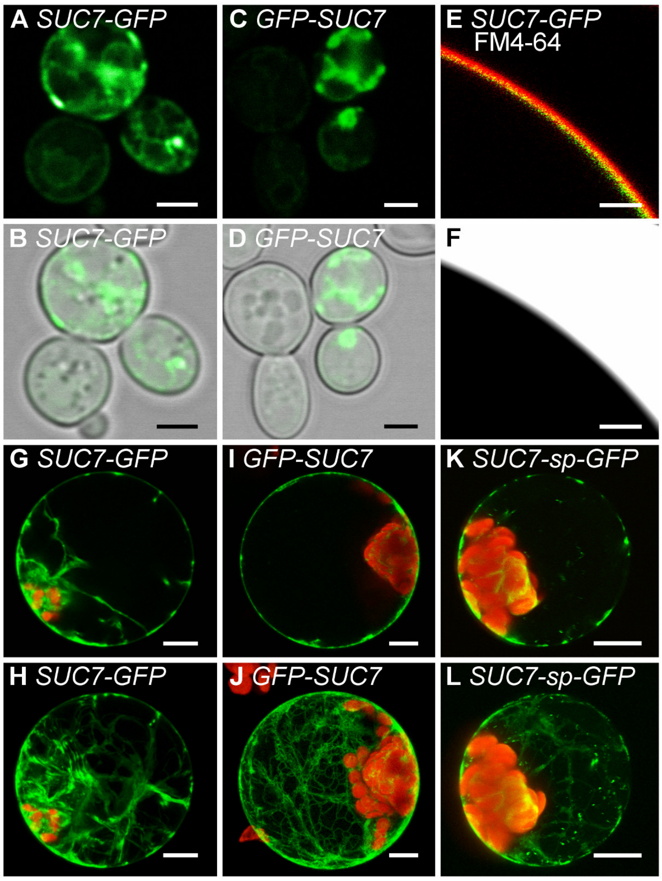

Supplementary Figure 2: **Subcellular localization of AtSUC7$_{Col\text{-}0}$ in different expression systems.** **(A)** Confocal image of yeasts expressing *AtSUC7-GFP*. **(B)** Bright field overlay to A. **(C)** Confocal image of yeasts expressing *GFP-AtSUC7*. **(D)** Bright field overlay to C. **(E)** Confocal image of a *Xenopus laevis* oocyte expressing *AtSUC7-GFP*. FM4-64 (red) labeled the plasma membrane and did not colocalize with AtSUC7-GFP. **(F)** Bright field to E. **(G-L)** Confocal images of Arabidopsis mesophyll protoplasts transformed with the indicated *GFP* fusion constructs of *AtSUC7* under the control of the *35S* promoter. sp = spacer. GFP is given in green, chlorophyll autofluorescence in red. **(G, I, K)** Single optical sections. **(H, J, L)** Maximum projections. Scale bars: 2.5 µm in A-D; 50 µm in E and F; 10 µm in I-L.

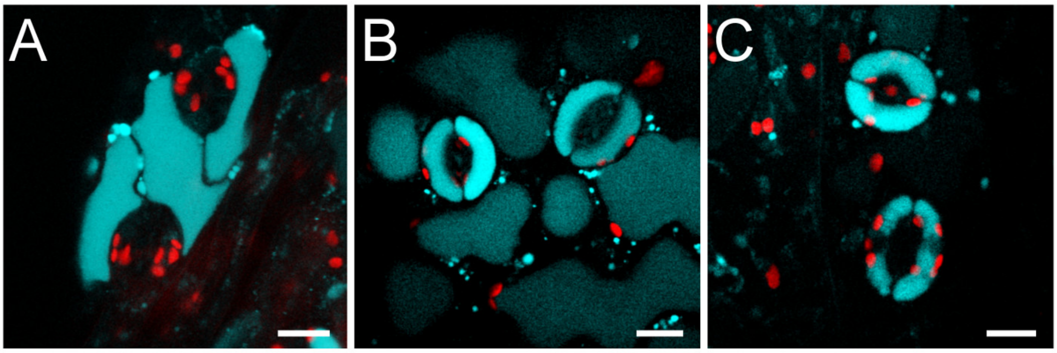

Supplementary Figure 3: **Analysis of esculin uptake into epidermis cells of the stomatal complex.** Epidermal peels of Col-0 leaves of different developmental stages were incubated with 1-mM esculin in W5 for 1 h prior to confocal microscopy. Esculin is given in cyan, chlorophyll fluorescence in red. **(A)** Small leaf (<3 mm) with esculin uptake into subsidiary cells. **(B)** Medium-sized leaf (transition from sink to source) with esculin in subsidiary cells and guard cells. **(C)** Source leaf with esculin accumulating in guard cells only. Scale bars: 10 µm

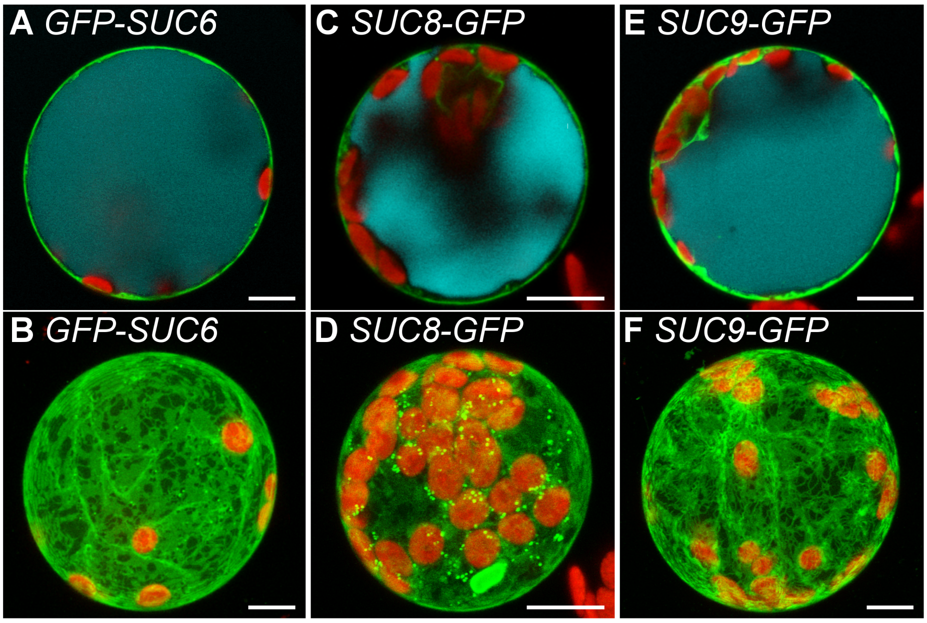

Supplementary Figure 4: **Confocal images of the subcellular localization of GFP-AtSUC6$_{\text{Col-0}}$, AtSUC8-GFP and GFP-AtSUC9 in Arabidopsis protoplasts**. **(A, C, E)** Single optical sections of mesophyll protoplasts expressing the indicated construct under the control of the *35S* promotor. **(B, D, F)** Maximum projections of protoplasts expressing the indicated constructs. GFP is shown in green, chlorophyll autofluorescence in red. Scale bars: 10 µm

Supplementary Table 3: Primers used for the detection of *AtSUC* transcripts in the T-DNA insertion lines *Atsuc6.3* and *Atsuc7.3* by RT-PCR.

Mutant line	Amplified allele	Primer name	Primer sequence
Atsuc6.3	upstream	AtSUC6c+9f	5'-ACCTCCAAGCAAACAAAGATGCAG-3'
		AtSUC6c+622r	5'-GCGCAATAGATGTCGCATGCTT-3'
	downstream	AtSUC6g+1364f	5'-CGTTCGCGCTAGCTTCCATAATC-3'
		AtSUC6c+1521r	5'-CATGTATGGGTCCTTATCTAGTGCTACAATAA-3'
	traversing	AtSUC6g+911f	5'-GTCGTGAGGTGTACGGTGGAGAC-3'
		AtSUC6g+1875r	5'-ACCACAAATCCTGGTAAATTCCCAC-3'
Atsuc7.3	upstream	AtSUC7-32f	5'-GCATATATAATAAATAAAACGCATAAACTTTTTAAGG-3'
		AtSUC7g+1107r	5'-GGAGCTGTGAATATTATTCTCGCCGTG-3'
	downstream	AtSUC7g+1787f	5'-CATGGCAATTGTCATACCACAAATG-3'
		AtSUC7c+1507r	5'-GCATATATAATAAATAAAACGCATAAACTTTTTAAGG-3'
	traversing	AtSUC7c+1032f	5'-TGGTATTGAAGGTATTAGTTAGGAAGATGGGA-3'
		AtSUC7c+1507r	5'-GCATATATAATAAATAAAACGCATAAACTTTTTAAGG-3'

Supplementary Table 4: List of primers used for generation of p*AtSUC:AtSUC*g-*GUS*, p*AtSUC:AtSUC*g-*GFP* and p*AtSUC7:GUS* reporter lines.

Gene	Primer name	Primer sequence
AtSUC6	AtSUC6-2547f+CACC	5'-CACCGTGGGAATTTACCAGGATTTGTGGT-3'
	AtSUC6c+1476r	5'-TGGTAAAACGGAAAATGCCACAAC-3'
AtSUC7	AtSUC7-1896f+CACC	5'-TATAGGTGCGGCAAAAACAAACCC-3'
	AtSUC7c+1473r	5'-GCAGTATTGTGGCATTTACCGTTTTACCT-3'
	AtSUC7-1896f+SbfI	5'-ACCTGCAGGTATAGGTGCGGCAAAAACAAACCC-3'
	AtSUC7-1r+AscI	5'-TGGCGCGCCCTTCTTCACCCTTGTCTTAGAGGTTG-3'

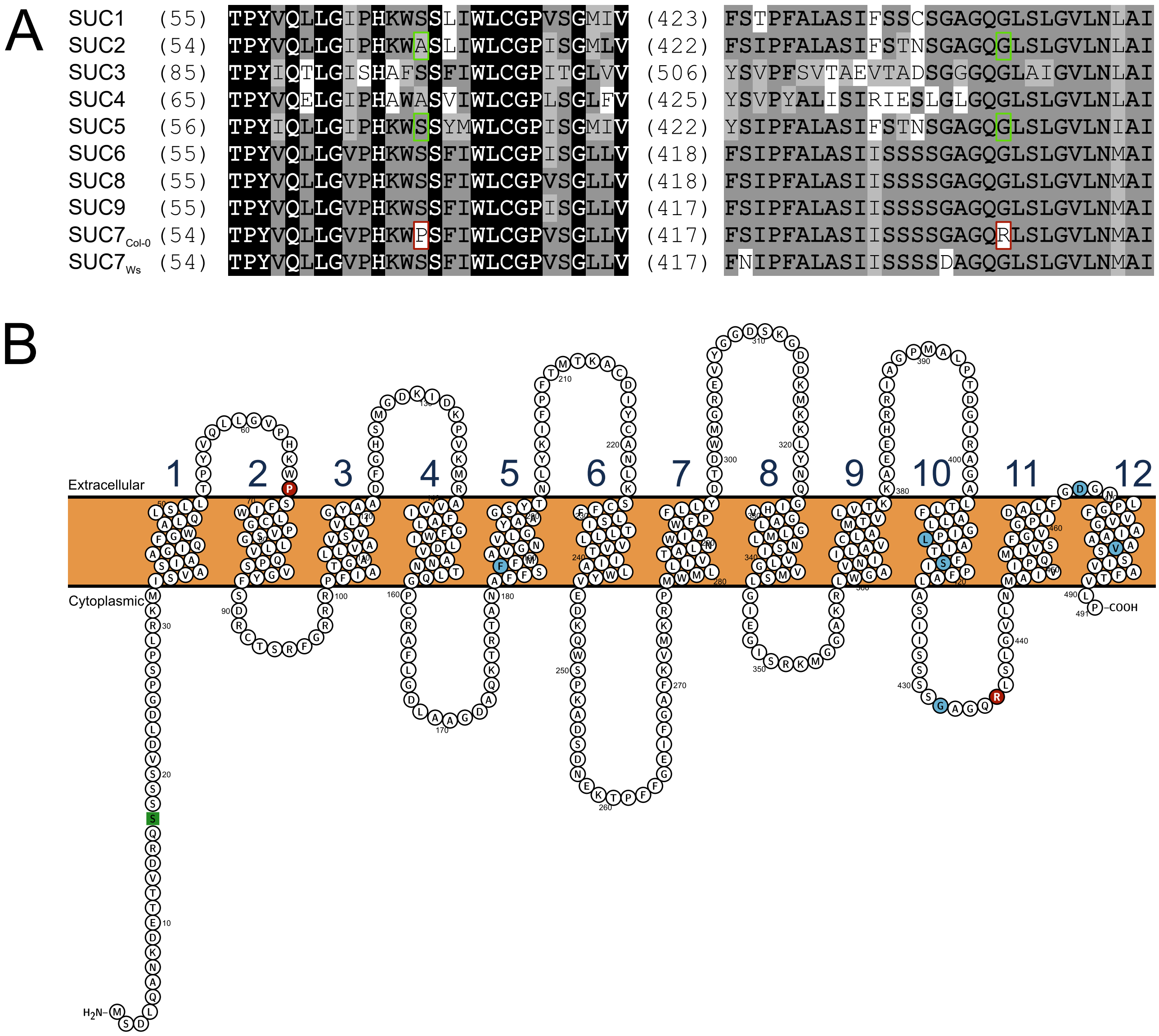

Supplementary Figure 5: **Sequence alignment of different Arabidopsis SUCs and predicted transmembrane structure of AtSUC7. (A)** Alignment of two regions of the Col-0 protein sequences of all Arabidopsis SUCs. The AtSUC7 sequence is additionally given for ecotype Ws. The two amino acids conserved in all AtSUCs but differing in AtSUC7$_{\text{Col-0}}$ are indicated by red rectangles. Green rectangles mark the amino acids point mutated in constructs AtSUC2$_{\text{A67P/G441R}}$ and AtSUC5$_{\text{S69P/G441R}}$. **(B)** Topological model of AtSUC7$_{\text{Col-0}}$ predicted and visualized using Protter (Omasits et al., 2013). Amino acids of AtSUC7$_{\text{Col-0}}$ differing from the conserved ones in all other AtSUCs are labeled in red. Additional amino acid exchanges compared to AtSUC7$_{\text{Ws}}$ are marked in blue.

Supplementary Table 5: List of primers used for amplification of *AtSUC6*$_{\text{Col-0}}$ and *AtSUC7*$_{\text{Col-0}}$ for the generation of constructs for heterologous expression in baker´s yeast.

Gene	Primer name	Primer sequence
AtSUC6	AtSUC6c+1f+YES+NotI	5’-TAGCGGCCGCAAGCTTGTAAAAGAAATGAGTGACCTCCAAGCAAACAAAG-3‘
	AtSUC6c+1479rev+NotI	5’-TAGCGGCCGCTTATGGTAAAACGGAAAATGCCACA-3‘
AtSUC7	AtSUC7c+1f+YES+NotI	5’-GCGGCCGCAAGCTTGTAAAAGAAATGAGTGACCTCCAAGCAAACAAAGATG-3‘
	AtSUC7c+1476r+NotI	5’-GCGGCCGCTTAAGGTAAAACGGTAAATGCCACAATACTG-3‘
	AtAtSUC7-5‘-BspHI-f	5’-TCATGAGTGACCTCCAAGCAAACAAAG-3‘
	AtAtSUC7-3‘-BspHI-r	5’-TCATGACAGGTAAAACGGTAAATGCC-3‘

Supplementary Table 6: List of primers used for generation of *AtSUC* fusion constructs with *GFP* for the expression in protoplasts. Names of the sequencing plasmids, destination vectors and resulting plasmids are listed in column 3, 4 and 5, respectively. If constructs were already published the list contains the name and reference of the respective construct. References: [1]Schneider et al., 2012; [2]Curtis and Grossniklaus, 2003; [3]Dotzauer et al., 2010; [4] Rottmann et al., 2016; [5]Feuerstein et al., 2010; [6]Klepek et al., 2005.

CDS	Primer name	Primer sequence	Sequencing plasmid	Destination vector	Plasmid name
AtSUC1	pAF30[5]	(AtSUC1c-pSO35e[6])			
AtSUC2	AtSUC2c+5'f+PciI	5'-ATACATGTCCAGCCATCCAATGGAG-3'	pJET1.2 blunt	pSB30[1]	pSS105
	AtSUC2c-3'r+PciI	5'-ATACATGTTGGCTGCGTGAAATCCCATAG-3'			
AtSUC2 **A67P/G441R**	AtSUC2c+5'f+PciI	5'-ATACATGTCCAGCCATCCAATGGAG-3'	pJET1.2 blunt	pSB30[1]	pTR307
	AtSUC2c+210r_SDM	5'-AATCAGAGAAGGCCATTTATGTGGG-3'			
	AtSUC2c+186f_SDM	5'-CCCACATAAATGGCCTTCTCTGATT-3'			
	AtSUC2c+1310f_SDM	5'-GTGCCGGCCAAAGACTTTCC-3'			
	AtSUC2c+1329r_SDM	5'-GGAAAGTCTTTGGCCGGCAC-3'			
	AtSUC2c-3'r+PciI	5'-ATACATGTTGGCTGCGTGAAATCCCATAG-3'			
AtSUC3	AtSUC3c+1f+CACC	5'-CACCATGAGTGACTCGGTGTCGATCTCG-3'	pENTR/D-TOPO	pMDC43[2]	pTR241
	AtSUC3c+1785r	5'-TTAGCCGATGTGGAAACCGGT-3'			
AtSUC5 **S69P/G441R**	AtSUC5c+1f+BspHI	5'-ATCATGAGTATGGGAGCCTTGGAAGCAGAA-3'	pSS87[1]	pTR302	
	AtSUC5c+214r_SDM	5'-TGTAAGACGGCCATTTGTGAGGG-3'			
	AtSUC5c+192f_SDM	5'-CCCTCACAAATGGCCGTCTTACA-3'			
	AtSUC5c+1309f_SDM	5'-GGTGCCGGCCAAAGACTTTC-3'			
	AtSUC5c+1328r_SDM	5'-GAAAGTCTTTGGCCGGCACC-3'			
	AtSUC5c+1536r+BspHI	5'-TATCATGACATGGAATCCCATAGCCCCTGAC-3'			
AtSUC6	AtSUC6c-54f	5'-TCATCTCCTCCCTCACCAACACC-3'	pJET1.2 blunt	pMDC43[2]	pFC12
	AtSUC6c+622r	5'-GCGCAATAGATGTCGCATGCTT-3'	pENTR/D-TOPO	pMDC83[2]	pFC13
	AtSUC6c+581f	5'-TGGGATATGCGGCCGGAT-3'			
	AtSUC6c+1521r	5'-CATGTATGGGTCCTTATCTAGTGCTACAATAA-3'			
	AtSUC6c+1fw+CACC	5'-CACCATGAGTGACCTCCAAGCAAACAAAGA-3'			
	AtSUC6c+1476rev+AscI	5'-GGCGCGCCTTGGTAAAACGGAAAATGCCACAAC-3'			
	AtSUC6c+1479rev+AscI	5'-GGCGCGCCTTTATGGTAAAACGGAAAATGCCACAAC-3'			
AtSUC7	AtSUC7-32f	5'-GCATATATAATAAATAAAACGCATAAACTTTTTAAGG-3'	pJET1.2 blunt	pSS87[1]	pTR58
	AtSUC7c+1507r	5'-GCATATATAATAAATAAAACGCATAAACTTTTTAAGG-3'		pCS120[3]	pTR57
	AtSUC7+1f+BspHI	5'-TCATGAGTGACCTCCAAGCAAACAAAG-3'			
	AtSUC7c+1473r+BspHI	5'-TCATGACAGGTAAAACGGTAAATGCC-3'			
AtSUC7 **-spacer**	AtSUC7c+1f+CACC	5'-CACCATGAGTGACCTCCAAGCAAACAAAGA-3'	pENTR/D-TOPO	pMDC43[2]	pTR72
	AtSUC7c+1473r	5'-GCAGTATTGTGGCATTTACCGTTTTACCT-3'		pMDC83[2]	pTR73
	AtSUC7c+1476r	5'-TTAAGGTAAAACGGTAAATGCCACAATACT-3'			
AtSUC7 **Ws**	AtSUC7c+1f+CACC	5'-CACCATGAGTGACCTCCAAGCAAACAAAGA-3'	pENTR/D-TOPO	pMDC43[2]	pTR253
	AtSUC7c+1507r	5'-GCATATATAATAAATAAAACGCATAAACTTTTTAAGG-3'			
AtSUC7 **P67S**	AtSUC7c+1f+CACC	5'-CACCATGAGTGACCTCCAAGCAAACAAAGA-3'	pJET1.2 blunt	pMDC43[2]	pTR244
	AtSUC7c+203r_SDM	5'-GATGACCATTTGTGTGGGACTC-3'	pENTR/D-TOPO		
	AtSUC7c+182f_SDM	5'- GAGTCCCACACAAATGGTCATC-3'			
	AtSUC7c+1476r	5'-TTAAGGTAAAACGGTAAATGCCACAATACT-3'			
AtSUC7 R436G	AtSUC7c+1f+CACC	5'-CACCATGAGTGACCTCCAAGCAAACAAAGA-3'	pJET1.2 blunt	pMDC43[2]	pTR245
	AtSUC7c+1315r_SDM	5'-GAGAAAGTCCTTGACCGGCGC-3'	pENTR/D-TOPO		
	AtSUC7c+1295f_SDM	5'-GCGCCGGTCAAGGACTTTCTC-3'			
	AtSUC7c+1476r	5'-TTAAGGTAAAACGGTAAATGCCACAATACT-3'			

AtSUC7 **P67S/R436G**	AtSUC7c+1f+CACC<br>AtSUC7c+1476r	5’-CACCATGAGTGACCTCCAAGCAAACAAAGA-3‘<br>5’-TTAAGGTAAAACGGTAAATGCCACAATACT-3‘	pENTR/D-TOPO	pMDC43[2]	pTR181
AtSUC8	AtSUC8c+1f+CACC<br>AtSUC8c-3’r-wob	5’-CACCATGAGTGACCTCCAAGCAAAAAAACG-3‘<br>5’-GGATCMAGGTAACACGGTAAATGC-3‘	pENTR/D-TOPO	pMDC43[2]<br>pMDC83[2]	pND100<br>pND101
AtSUC9	AtSUC9c+1f+BspHI<br>AtSUC9c+1473r+BspHI	5’-TCATGAGTGACATCCAAGCAAAAGAAG-3‘<br>5’-TCATGACAGGTAAAACGGTAAGTGCC-3‘	pJET1.2 blunt<br>pCR-Blunt II-TOPO	pSS87[1]<br>pCS120[3]	pTR318<br>pND98
STP10	pTR147[4]	(STP10c-pMDC43[2])			
SWEET4	SWEET4c+1f+NcoI<br>SWEET4c+753r+NcoI	5’-ATGGTTAACGCTACAGTTGCGAGAAA-3‘<br>5’-GTGGACAAGCTAAACGAGTTTCAGCT-3‘	pJET1.2 blunt	pCS120[3]	pTR264
SWEET10	SWEET10c+1f+NcoI<br>SWEET10c+867r+NcoI	5’-ATCCATGGCAATTTCACAAGCCGTCTT-3‘<br>5’-ATCCATGGCATTCTTAGAAATGAGAAATACTTCTTTTTCATCC-3‘	pJET1.2 blunt	pCS120[3]	pTR311

Literature cited

Curtis, M. D., and Grossniklaus, U. (2003). A Gateway cloning vector set for high-throughput functional analysis of genes in planta. *Plant Physiol* 133, 462–469

Dotzauer, D., Wolfenstetter, S., Eibert, D., Schneider, S., and Sauer, N. (2010). Novel PSI domains in plant and animal H^+-inositol symporters. *Traffic* 11, 767–781

Feuerstein, A., Niedermeier, M., Bauer, K., Engelmann, S., Hoth, S., Stadler, R., and Sauer, N. (2010). Expression of the *AtSUC1* gene in the female gametophyte , and ecotype-specific expression differences in male reproductive organs. *Plant Biol* 12, 105–114

Klepek, Y.-S., Geiger, D., Stadler, R., Klebl, F., Landouar-Arsivaud, L., Lemoine, R., Hedrich, R., and Sauer, N. (2005). Arabidopsis POLYOL TRANSPORTER5, a new member of the monosaccharide transporter-like superfamily, mediates H^+-symport of numerous substrates, including myo-inositol, glycerol, and ribose. *Plant Cell* 17, 204–218

Omasits, U., Ahrens, C. H., Müller, S., and Wollscheid, B. (2014). Protter: interactive protein feature visualization and integration with experimental proteomic data. *Bioinformatics* 30, 884-886

Rottmann, T., Zierer, W., Subert, C., Sauer, N., and Stadler, R. (2016). *STP10* encodes a high-affinity monosaccharide transporter and is induced under low-glucose conditions in pollen tubes of Arabidopsis. *J Exp Bot* 67, 2387–2399

Schneider, S., Hulpke, S., Schulz, A., Yaron, I., Höll, J., Imlau, A., Schmitt, B., Batz, S., Wolf, S., Hedrich, R., et al. (2012). Vacuoles release sucrose via tonoplast-localised SUC4-type transporters. *Plant Biol* 14, 325–336
